# Co-release of opposing signaling molecules controls the escalation and release of aggression

**DOI:** 10.1101/2025.03.13.643119

**Published:** 2025-06-08

**Authors:** Rachel E. Gatlin, Jordyn Gagon, Damhyeon Kwak, Sewon Park, Hailee Walker, Lo Kronheim, Thomas Everett, Ashley Covington, Michaela M. Fluck, Tobias Zickmund, Nicholas A. Frost, Moriel Zelikowsky

**Affiliations:** 1. Howard Hughes Medical Institute; 2. Department of Neurobiology, University of Utah, School of Medicine, Salt Lake City, UT, 84112.; 3. Department of Neurology, University of Utah, School of Medicine, Salt Lake City, UT, 84132.; 4. The Neuroscience Program, University of Utah, School of Medicine, Salt Lake City, UT, 84112.

## Abstract

Neuropeptides exert broad effects across the brain to influence behavior. However, the mechanism by which the brain uses neuropeptide signaling to exert top-down control over complex behavioral responses remains poorly understood. Prolonged social isolation induces a distinct internal state that results in sweeping changes to behavior, including increased aggression. Here, we find that isolation-induced aggression activates Tachykinin-2 expressing (Tac2^+^) neurons in the mouse medial prefrontal cortex (mPFC). Genetic characterization of Tac2^+^ cells in the mPFC reveals them to be a population of unexplored GABAergic neurons. In-depth behavioral analyses combined with *in vivo* recordings of neural activity demonstrate that mPFC Tac2^+^ neurons are tuned to distinct phases of isolation-induced aggression: investigatory behaviors that escalate to attack (aggression escalation) and attack itself (aggression release). Loss-of-function perturbations targeting the release of either Neurokinin B (NkB), the stimulatory peptide encoded by *Tac2*, or the inhibitory neurotransmitter, GABA, reveal that these signaling molecules exert dissociable control over aggression escalation and release. These findings identify distinct roles for opposing signaling molecules co-released from the same population of cells. This suggests a surprising neurochemical mechanism by which neuropeptidergic populations in the prefrontal cortex exert top-down influence over complex behavior via the co-release of signaling molecules with opposing actions but coordinated impacts on behavior.

## INTRODUCTION

Neuropeptides are thought to play a privileged role in the control of internal states. Their extended time course for signaling through G-protein coupled receptors (GPCRs) combined with their scalability of release magnitude and diffusion over long distances leaves them well poised to modulate long-lasting states ^1–5^. In *C. elegans,* distinct circuits comprised of antagonistic neuromodulators allow for rapid behavioral state transitions (Flavell et al., 2013) and shape the acquisition of adaptive behavior ^6^. In mice, subcortical neurons utilize opposing signaling molecules to similarly guide behavioral state transitions ^7^and enable the downstream convergence of multiple signals to drive a single behavior ^8^. However, much of our understanding of neuropeptidergic control over internal states and behavior has been driven by studies in subcortical regions ^8–11^. In contrast, little is known about the general contributions of neuropeptide signaling in the cortex and how these interact with classical neurotransmitter signaling systems to control behavior.

The medial prefrontal cortex (mPFC) is a social executive center in the brain. The mPFC combines external information about an animal’s environment and its social setting with internal information about an animal’s social history and current state to coordinate behavioral outputs via “top-down” projections to downstream structures ^12–18^. Output of the mPFC is controlled through a careful balance of excitation and inhibition, which is highly sensitive to changes in experience, such as stress ^19–21^. While the mPFC is thought to exert executive control over complex behaviors, the signaling mechanisms which underlie this control, particularly in states of stress, remain poorly understood.

One key source of stress known to impact the mPFC is social isolation (SI). Indeed, extended periods of social isolation have been shown to generate negative alterations in social behavior, including increased aggression, via changes to mPFC activity profiles ^9,22–27^. Despite this, the identity of genetically defined populations of mPFC neurons and their functional influence over isolation-altered behaviors has been relatively unexplored. In particular, the role that neuropeptidergic populations within the mPFC play in controlling the internal state induced by prolonged SI is unclear.

The Tachykinin-2 (Tac2) neuropeptide system has emerged as an example of subcortically distributed, neuromodulatory control over the state induced by prolonged isolation ^9^. Previous work has further implicated subcortical Tac2 neurons in emotional and social behaviors ^9,28–35^. This role for the Tachykinin system seems to be evolutionarily conserved, as similar findings have been identified in *Drosophila* ^34,36^. While *Tac2* is known to be expressed in the mPFC, its cortical function remains entirely unexplored.

Here, we target the Tac2 system to interrogate the mechanism by which neuropeptides in the mPFC exert control to influence complex behavior. Using in-depth behavioral and genetic analyses, single nucleus RNA-sequencing (snRNAseq), cell-type-specific recordings and perturbations, and CRISPR-mediated mutagenesis, we identify Tac2^+^ neurons in the mPFC as being a population of unexplored GABAergic neurons whose activity is required for isolation-induced aggression. We find that Tac2^+^ mPFC cells are tuned to two distinct phases of isolation-induced aggression: investigatory behaviors that escalate to attack (aggression escalation) and attack behaviors themselves (aggression release). Molecule-specific loss-of-function perturbations targeting the ability of Tac2^+^ mPFC neurons to release either Neurokinin B (NkB), the stimulatory peptide encoded by *Tac2*, or the inhibitory neurotransmitter GABA, reveal that these signaling molecules exert dissociable control over aggression escalation and release, respectively. Collectively, these results highlight a crucial role for neuropeptidergic signaling in the mPFC in the modulation of internal states and reveal that surprisingly, the coordinated execution of state-induced behaviors occurs via action of opposing signaling molecules.

## RESULTS

### Prolonged social isolation induces a state of aggression comprised of attack behavior and social behaviors escalating to attack

To determine the contribution of cortical neuropeptide signaling to internal state control, we leveraged our model of chronic social isolation, which induces a state characterized by altered social behavior and increased aggression in male mice ^9,22–25,37,38^. While social isolation is well studied in males, it remains unclear how females are impacted by prolonged isolation, though recent work suggests that prolonged (Pinna, Dong et al. 2003, Morè 2008, Tan, Wang et al. 2021) and acute periods of social isolation in females are sufficient to reshape prosocial behavior ^39^

**Figure 1.**
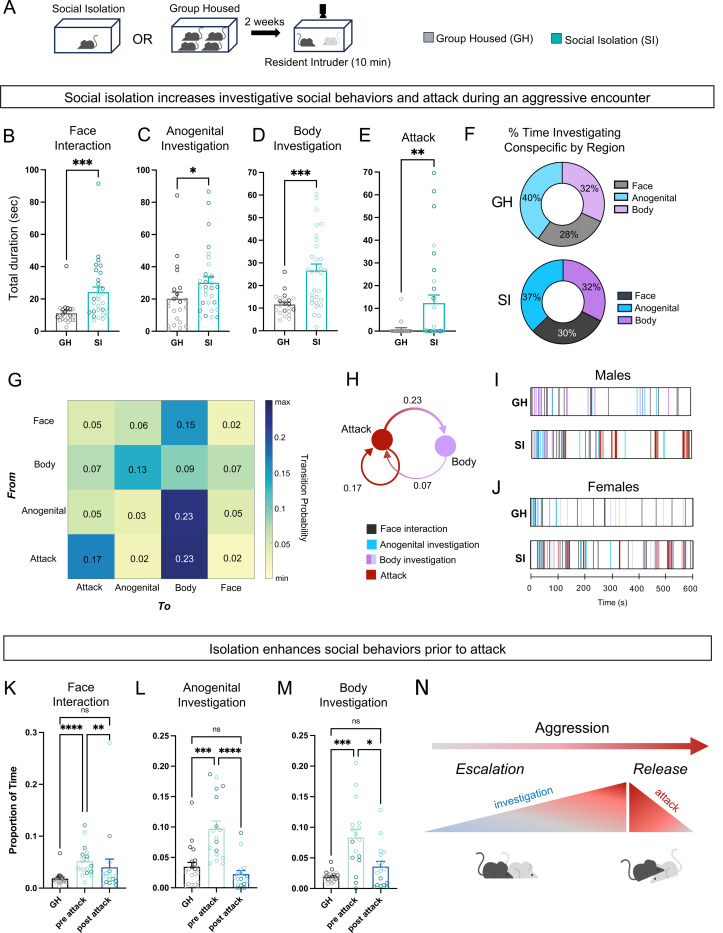
Social isolation induces aggression escalation and attack in males and females. **A**. Diagram showing the workflow for the Resident Intruder assay in group housed and isolated mice. **B**. Total duration of face interaction in group housed (GH) and isolated (SI) mice. Mann-Whitney test, p = 0.001 **C**. Same as B, but anogenital investigation. Mann-Whitney test p < 0.05 **D**. Same as B, but body investigation. Unpaired t-test p = 0.0001 **E**. Same as E, but attack. Mann-Whitney test p < 0.01 **F**. Percent of time investigating conspecific by body region (face, body, anogenital) in group housed and isolated mice. Time investigating region / total investigation time x 100). **G**. Transition matrix showing probability of isolated animal transitioning from behaviors to other behaviors. **H**. Diagram showing most common behavior transitions from G. **I**. Representative raster plots from a group housed (GH) and an isolated (SI) male during the Resident Intruder assay. **J**. Same as I, but in females. **K**. Proportion of time group housed (GH) and isolated mice spend engaged in face interaction before and after the first instance of attack. Kruskal-Wallis test p < 0.001, Dunn’s multiple comparisons test **L**. Same as L, but anogenital investigation. Kruskal-Wallis test p < 0.0001, Dunn’s multiple comparisons test **M**. Same as K, but body investigation. Brown-Forsythe ANOVA p < 0.0001, Dunnett’s multiple comparisons test. **N**. Diagram depicting isolated animals transition towards attack in the Resident Intruder Assay Bars represent mean ± SEM, dots within bars represent mean behavior duration for each animal * p < 0.05, ** p < 0.01, *** p < 0.001, **** p < 0.0001 Corresponds to S1

To examine the impacts of prolonged social isolation (SI) across both sexes, male and female mice were isolated for two weeks and tested on the resident intruder (RI) assay ^40^ (Fig 1A). Compared to group housed controls (GH), socially isolated (SI) mice displayed an increase in the duration of time spent engaged in social behaviors directed towards a novel conspecific mouse, including face interaction, anogenital investigation, and body investigation (Fig 1B–D) ^41^. These effects were maintained across investigation categories, demonstrating that isolation produced a global increase in social investigatory behaviors, rather than a re-shuffling of how to allocate these behaviors (Fig 1F). In addition, SI mice showed a significant increase in attack behavior (Fig 1E), consistent with prior studies ^9,23,34,42^. Importantly, these effects were detected in both males and females (Fig. S1A–E, S1K–O), suggesting that prolonged social isolation shifts mice into a state of increased aggression, regardless of sex.

To systematically assess how social isolation reshapes behavior, we generated behavioral transition matrices to determine the probably of transiting from one behavior to the next in SI animals ^43–45^. SI mice were most likely to transition from attack bouts to body investigation and back, or from one attack bout into another (Fig G-H), suggesting that once attack is “released,” attack begets attack. Interestingly, behavioral raster plots visualizing investigatory and attack bouts revealed that once attack began, investigatory behaviors were rarely observed after the initiation of attack in males or females (Fig 1I–J), suggesting that the increased investigatory behavior observed in SI mice only occurs in the behavioral window leading up to attack.

To further explore whether the onset of attack inhibits investigation behaviors in SI mice, we examined the proportion of time aggressive SI mice engaged in investigation before and after the first attack bout compared to GH mice (i.e. non-fighters). We found that indeed, following the initial onset of attack, enhanced social investigation in SI mice was significantly reduced, bringing them down to levels equivalent to those of GH mice (Fig 1 K–N; Fig S1 H–J; Fig S1 R–T). These findings suggest that social isolation induces a state of aggression comprised of an initial escalatory phase in which social investigatory behaviors are elevated (“aggression escalation”), which is then replaced by a second phase in which aggression is released, and animals switch to attack behaviors (“aggression release”) (Fig 1N).

### Tac2^+^ mPFC neurons are recruited during isolation-induced aggression

**Figure 2.**
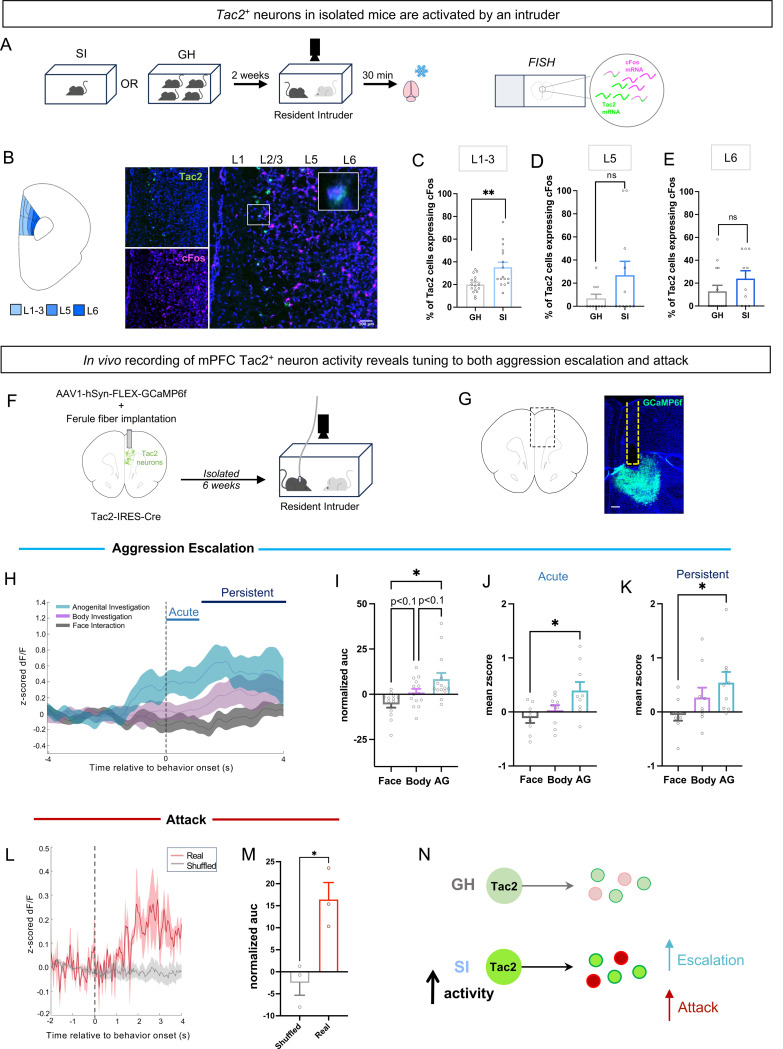
Prefrontal Tac2^+^ neural activity is tuned to isolation-induced aggression **A**. Workflow to perform fluorescent in situ hybridization (FISH) on mPFC sections from isolated (SI) and group housed (GH) mice. **B.** Diagram showing distribution of cortical layers in mouse mPFC. Representative image of FISH for *Tac2* (green) and *cFos* (pink), scale bar represents 100 μm. **C**. Percent of Tac2 cells expressing cFos in layers 1–3; nested t-test p < 0.01. **D**. Percent of Tac2^+^ cells expressing *cFos* in layer 5; nested t-test p > 0.05. **E**. Percent of Tac2^+^ cells expressing cFos in layer 6; nested t-test, p > 0.05. **F**. Workflow of fiber photometry experiments. **G**. Representative image of fiber tract and GCaMP6f expression (green). Scale bar represents 100μm. **H.** Z-scored change in fluorescence (dF/F) of GCaMP with 0.5s Gaussian smoothing at the onset of (0) through 4s after the experimental animal engages in face investigation, body investigation or anogenital investigation. **I**. Normalized area under the curve of dF/F across 0–4s, dots within bars represent mean for individual animals; Friedman test p < 0.0001, Dunn’s multiple comparison’s test **J**. Acute (1s) response (mean z-score of dF/F) of mPFC Tac2 neurons during face interaction, body investigation and anogenital (AG) investigation; one-way ANOVA p < 0.05, Tukey’s multiple comparisons test. **K**. Persistent (3s) Tac2 neuron responses (mean z-score of dF/F) during face interaction, body investigation and anogenital (AG) investigation; One-way ANOVA p < 0.05, Tukey’s multiple comparison’s test. **L**. mPFC Tac2 neuron responses (z-scored dF/F) at the onset of and during attack bouts (“real,” red) versus shuffled Tac2 responses (“shuffled,” grey). **M**. Bar graph of normalized area under the curve of dF/F during attack vs scrambled control; unpaired t-test p < 0.05. **N**. Proposed model for Tac2 neuronal activity contribution to escalation and attack in SI mice. Bars represent mean ± SEM, * p<0.05 ** p<0.01, dots in bars for FISH represent counts of co-localization in each section, dots in bars in fiber photometry data represent mean response in each animal. Corresponds to S2

We previously identified a role for subcortical *Tac2/*NkB signaling in the control of social isolation ^9^. Recent work has implicated the mPFC in the control of aggression ^21,22^ but the neurochemical mechanism within the cortex which enables social isolation to convert animals to a state of aggression is unknown. To determine whether isolation-induced aggressive encounters engage mPFC Tac2^+^ neurons in isolated mice, we assessed whether Tac2^+^ cells were activated following the RI assay in male and female SI mice. Using RNAscope to probe for *Tac2* and the activity marker *cFos,* we found a significant increase in cFos-expressing, early layer Tac2^+^ neurons in SI mice compared to GH controls (Fig 2A–C).

As cFos expression cannot disentangle which behaviors Tac2^+^ cells were activated during, we performed fiber photometry recordings to determine the *in vivo* activity of mPFC Tac2^+^ neurons in SI male and female mice during the Resident Intruder assay (Fig 2F–G). Tac2-IRES-Cre mice ^9,46^ were injected with a virus encoding a Cre-dependent calcium indicator (GCaMP6f) and implanted with a ferule fiber targeting the mPFC to enable *in vivo* recordings of Tac2^+^ population activity following social isolation. Neural activity during the RI assay was aligned to three distinct investigatory behaviors which comprise the escalation to aggression: face interaction, body investigation, and anogenital (AG) investigation. Interestingly, we found that neural activity (dF/F) was significantly increased during bouts of anogenital investigation and trending upwards for body investigation when compared to face interaction, suggesting that Tac2^+^ neurons are tuned to anogenital and body investigation, but not face interaction (Fig 2H–I). This effect persisted (3s) past the acute period (1s) immediately triggered by bout initiation (Fig 2J–K), consistent with a role for neuropeptides in long-lasting states ^3,4^. These data suggest that while isolation increases face interaction (Fig 1B), this behavior is not encoded by Tac2^+^ neurons in the mPFC.

To understand the response profile of these cells during attack behavior, we compared the activity of mPFC Tac2^+^ neurons in isolated aggressive animals during attack bouts to a control scrambled signal from the same animals (Fig 2L). Tac2^+^ neurons increase their activity at the onset of attack, which persists for several seconds (Fig 2M). Together, these data show that mPFC Tac2^+^ neurons are tuned towards both the escalation of aggression (investigatory behavior) and its release (attack behavior) (Fig 2N).

### Prefrontal Tachykinin-2 neurons represent a unique subclass of GABAergic neurons

**Figure 3.**
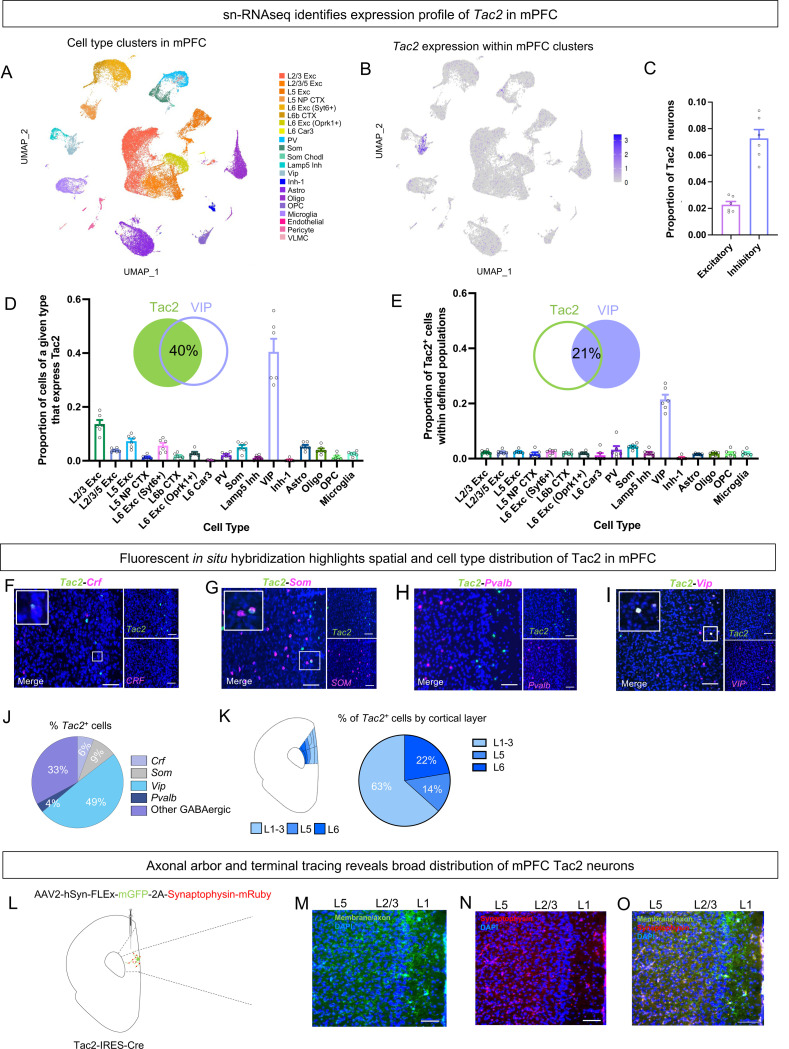
Prefrontal Tac2 neurons represent a population of GABAergic neurons that are active during social encounters. **A**. UMAP plot showing clusters of defined cortical cell types. **B**. UMAP plot showing clusters of defined of Tac2^+^ neurons within other defined cortical cell types. **C.** Proportion of excitatory and inhibitory neurons that express *Tac2*. **D**. Proportion of cells within a given genetically defined class that express *Tac2*. **E**. Proportion of Tac2^+^ cells that express a marker of a given genetically defined class. **F-I**. Representative images of RNAscope^™^ fluorescent in situ hybridization. scale bar represents 100μm. *Tac2*: Tachykinin-2, *Crf*: corticotropin releasing factor, *Som*: somatostatin, *Pvalb*: parvalbumin, *Vip*: Vasoactive intestinal peptide. **J**. Pie chart showing percent of Tac2^+^ cells expressing *Crf*, *Som, Pvalb*, and *Vip*. **K**. Diagram showing prelimbic and infralimbic regions of medial prefrontal cortex with cortical layers. L1–3: layers 1–3, L5: layer 5, L6: layer 6. Pie chart showing the percent of Tac2^+^ cells in cortical layers. **L**. Viral infusion into infralimbic mPFC. **M**. Representative image showing Tac2^+^ neuronal axons labeled with GFP and the cortical layers. **N**. Representative image of Tac2^+^ neuronal terminals (synaptophysin) in red (mRuby). **O**. Merged image showing axonal arbor (green) and terminals (red) of Tac2 neurons. **M-O**. scale bar represents 100μm. All bars are mean ± SEM, dot within bars represent mean for individual animals. Corresponds to S2

Previous research has found that social isolation decreases the spiking activity of pyramidal neurons in the PFC during aggression (Tan et al 2021) and increases mPFC GABAergic neuron activity ^23,47^. We found that social isolation increases the activity of Tac2^+^ neurons in the mPFC (Fig 2). Thus, we hypothesized that Tac2 labels an opposing population of neurons in the mPFC that express the inhibitory neurotransmitter GABA. To characterize the genetic profile of *Tac2*-expressing cells in the mPFC we used single nucleus RNA sequencing (sn RNAseq) as well as fluorescent in situ hybridization (RNAscope) ^48^. Our snRNAseq analyses revealed that *Tac2* is predominately expressed in inhibitory neuronal populations (Fig 3A–E, S2 A–C), consistent with findings in other cortical tissue ^49,50^, and that *Tac2* is minimally expressed glia (Fig 3A–D). RNAscope experiments confirmed that Tac2^+^ cells represents an almost entirely GABAergic population (*Gad1 and Gad2*^+^), with virtually no overlap with glutamatergic (*Slc17a7*^*+*^) cells (Fig S2A–C).

The largest gene to be co-expressed with *Tac2* was *vasoactive intestinal peptide* (*VIP*), which accounted for roughly half of Tac2^+^ neurons (Fig 3D). In turn, *Tac2* was present in roughly 22% of VIP^+^ cells (Fig 3E). Using RNAscope, we confirmed that Tac2^+^ neurons comprise a mix of distinct, genetically defined cell types, including those co-expressing *VIP* (Fig 3F–J), consistent with our sequencing data. This approach allowed us to assess the spatial profile of *Tac2* expression, where we found that the majority of Tac2^+^ neurons reside in layers 1–3 of cortex (Fig 3K), where they overlap with *VIP* by 60% (Fig S2D). However, unlike Tac2^+^ cells, VIP^+^ cells were not activated by the resident intruder assay following social isolation (Fig S2E), nor were Tac2^+^ cells that co-expressed *VIP* preferentially activated (Fig S2F–J), suggesting that VIP does not underlie NkB’s role in isolation-induced aggression (Fig 2).

Of interest, *Tac2* was not co-expressed with *Tacr3*, the gene encoding the Tac2-specific Neurokinin 3 receptor (Nk3R) (Fig S2K), suggesting that the tachykinin system exerts its influence on a dissociable population of *Tacr3*-expressing cells. This is supported by our finding that Tacr3^+^ cells in the mPFC are also activated by the resident intruder assay following social isolation (Fig S2L–O). These data suggest that Tac2^+^ cells represents a genetically unique class of neurons, predominately localized to the early cortical layers, that is activated by isolation-induced aggression.

To characterize the anatomical features of Tac2^+^ neurons in the mPFC, we visualized the axonal arbor of these cells using anterograde tracing. We expressed a Cre-dependent adeno-associated virus expressing membrane-bound GFP and synaptophysin fused to mRuby ^51^ in the mPFC of Tac2-Cre mice (Fig 3L). This tracing revealed Tac2^+^ cell bodies primarily reside in layers 1–3, with axonal arbors extending into layer 5 of mPFC and terminals spread throughout the cortical layers, with the highest density in layer 5 (Fig 3 M–O). These results illustrate that though sparse, this class of neurons has the potential to reach broadly across the mPFC to alter social behavior, consistent with the idea a small handful of neurons are sufficient to maintain a persistent state representation ^52^.

### mPFC Tac2^+^ neurons are necessary for isolation-induced attack

**Figure 4.**
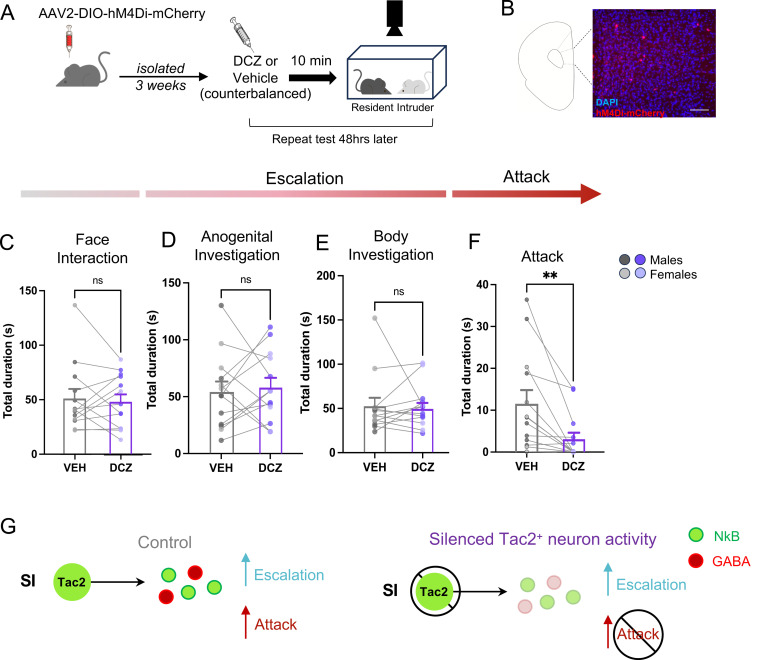
mPFC Tac2^+^ neuron activity is required for isolation-induced aggression. **A**. Experimental approach for DREADD-mediated inhibition of mPFC Tac2^+^ neurons. **B**. Representative image of hM4Di-mCherry labeled Tac2^+^ neurons in infralimbic mPFC, scale bar represents 100 μm. **C-F**. Effects of DREADD-mediation inhibition of mPFC Tac2^+^ neurons in mice given vehicle (VEH) or DCZ (Deschloroclozapine). **C**. Total duration of face threat: Wilcoxon test, p > 0.05. **D**. Total duration of anogenital investigation: Wilcoxon test, *p* > 0.05. **E**. Total duration of body investigation: Paired t-test, p > 0.05. **F**. Total duration of aggression: Wilcoxon test, *p* < 0.01. **G**. Models of Tac2 neuronal activity controlling aggression through release of signaling molecules. Bars represent mean ± SEM, * p<0.05 ** p<0.01, dots with connecting lines show mean of individual animal’s behavior duration on each drug. Corresponds to S3 and S4.

Given that Tac2^+^ neurons in the mPFC are tuned to isolation-induced aggression (Fig 2), we next tested whether the activity of mPFC Tac2^+^ neurons is necessary for aggression escalation or attack (Fig 4A). A Cre-dependent, virally encoded inhibitory DREADD virus ^53^(AAV2-hSyn-DIO-hM4D-mCherry) was injected into the mPFC of isolated, male and female Tac2-Cre mice (Fig 4B) to reversibly silence Tac2^+^ neurons. Mice were then tested on the RI assay twice – once under the influence of the DREADDs-activating ligand, Deschloroclozapine (DCZ, 0.1mg/kg) ^54^, and once under the influence of saline (order counter-balanced). Our DCZ dose had no off-target effects on behavior (Fig S3) and was sufficient to reduce mPFC Tac2^+^ activity (Fig S4I–J). Inhibiting mPFC Tac2^+^ neuron activity had no effect on face interaction or escalatory behaviors (body and anogenital investigation) (Fig 4C–E). However, SI-induced attack was significantly reduced when mPFC Tac2^+^ neurons were inhibited (Fig 4F). This effect was observed across both sexes (Fig S4 A–H), demonstrating that in mPFC, Tac2^+^ neuron activity is necessary for the effects of isolation to induce aggression in mice.

### *Tac2* knockdown in mPFC attenuates aggression escalation

**Figure 5.**
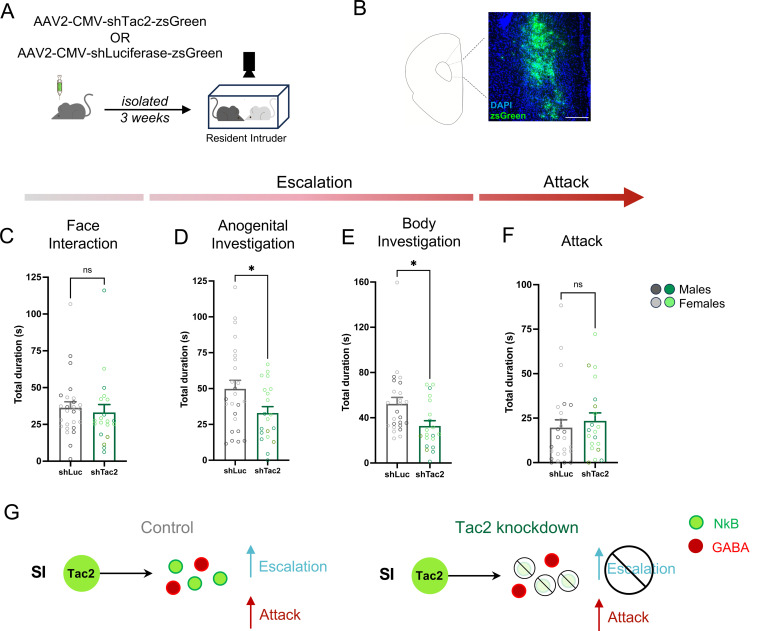
Knockdown of Tac2 in mPFC of isolated mice attenuates investigative behaviors that escalate to attack. **A**. Experimental approach for knockdown of *Tac2* in mPFC and subsequent behavioral testing. **B**. Image showing viral infusion site for shRNA virus in the mPFC **C**. Total duration of face threat in Luciferase shRNA mice (shLuc) and Tac2 shRNA mice (shTac2), Mann-Whitney test, *p* > 0.05. **D**. Total duration (s) of anogenital investigation in Luciferase shRNA mice (shLuc) and Tac2 shRNA mice (shTac2), unpaired t-test, *p* < 0.1. **E**. Total duration (s) of body investigation in Luciferase shRNA mice (shLuc) and Tac2 shRNA mice (shTac2), Mann-Whitney test, *p* < 0.05. **F**. Total duration of aggression in Luciferase shRNA mice (shLuc) and Tac2 shRNA mice (shTac2), Mann-Whitney test, p > 0.05. **G**. Diagrams depicting contributions of Neurokinin B (NkB) to escalation in isolated animals. Bars represent mean ± SEM, individual dots represent mean duration for each animal, ns p > 0.05, * p < 0.05 Corresponds to S5

Tac2^+^ neuronal activity is required for the effects of isolation to induce attack (Fig 4), but the neurochemical mechanism underlying this effect is unknown. Our sequencing data identified Tac2^+^ neurons as GABAergic (Fig 3), suggesting at least two potential signaling molecules which could exert control over the effects of isolation to impact behavior: release of the stimulatory neuropeptide encoded by *Tac2*, Neurokinin B (NkB), or release of the inhibitory neurotransmitter GABA (Fig 5A). To determine the contributions of NkB signaling to isolation-induced aggression, we used a viral mediated shRNAi to knockdown *Tac2* in the mPFC of isolated mice (Fig 5B–C, Fig S5I) ^9^. Wildtype isolated male and female mice were infused with Tac2-shRNA or a control shRNA (targeting luciferase) into the mPFC and tested on the RI assay ^9^ (Fig 5B). Surprisingly, knockdown of *Tac2* in the mPFC of isolated mice resulted in a significant decrease in aggression escalation behaviors (body investigation and anogenital investigation) but had no impact on face interaction (Fig 5D–F), the isolation-induced social behavior that Tac2^+^ mPFC cells were not tuned to (Fig 2H–K). In contrast, *Tac2* knockdown had no impact on attack behavior (Fig 5G). These effects were consistent across sex (Fig S5). These data reveal that NkB signaling contributes to the increase in investigation behaviors that escalate up to attack but not attack itself. Interestingly, these data are in opposition to our DREADD-mediated silencing effects (Fig 4), suggesting that neuropeptide signaling can exert control over behaviors that are not sensitive to a DREADD-mediated approaches, potentially through long lasting impacts of neuropeptide signaling across longer windows of time (i.e. prolonged isolation).

### Disruption of GABA co-release from mPFC Tac2^+^ neurons blocks attack

**Figure 6.**
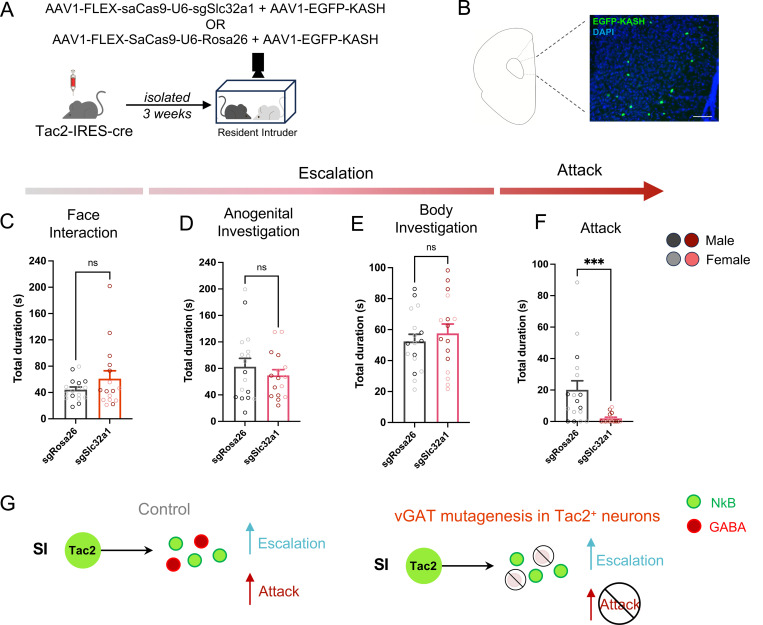
GABA release from mPFC Tac2^+^ neurons controls isolation-induced attack. **A**. Experimental workflow to infuse CRISPR viruses into mPFC of Tac2-IRES-Cre mice. **B**. Representative image of EGFP-KASH to visualize viral infusion site bar represents 100 µm. **C**. Total duration (s) of face threat in mice with guide RNA directed to the Rosa26 locus (sgRosa26) or Slc32a1 (sgSlc32a1), Mann-Whitney test, *p* > 0.05. **D**. Total duration (s) of body investigation in mice with guide RNA directed to the Rosa26 locus (sgRosa26) or Slc32a1 (sgSlc32a1), unpaired t-test, *p* > 0.05. **E**. Total duration (s) of body investigation in mice with guide RNA directed to the Rosa26 locus (sgRosa26) or Slc32a1 (sgSlc32a1), unpaired t-test, *p* > 0.05. **F**. Total duration (s) of aggression in mice with guide RNA directed to the Rosa26 locus (sgRosa26) or Slc32a1 (sgSlc32a1), Mann-Whitney test, *p* < 0.01. **G**. Proposed model of Neurokinin B and GABA release from mPFC Tac2 neurons to control distinct components of isolation-induced aggression (escalation and attack). Bars represent mean ± SEM, lighter dots within bars represent mean of individual female, darker dots male; ns p > 0.05, * p < 0.05, ** p < 0.01, *** p < 0.001 Corresponds to S6

Our loss-of-function results demonstrate that Tac2^+^ neuron activity is required for isolation-induced attack (Fig 4) but that this cannot be explained by release of NkB from these cells (Fig 5). To determine whether GABA transmission from mPFC Tac2^+^ neurons mediates isolation-induced attack, we used a CRISPR-based approach to generate loss-of-function mutations in *Slc32a1*, the gene encoding the vesicular GABA transporter (vGAT), in mPFC Tac2^+^ neurons. A viral cocktail including an AAV containing a Cre-dependent Staphylococcus aureus Cas9 (SaCas9) ^55^ and a guide RNA (sgRNA) directed to *Slc32a1* was injected into the mPFC of Tac2-Cre mice (Fig 6A–B) to induce non-sense mediated decay of *Slc32a1* in Tac2^+^ neurons only (Fig S6I). A vector expressing SaCas9 and a guide RNA directed to the Rosa26 locus ^56–58^, was injected in control mice. The CRIPSR virus was co-injected with a Cre-dependent cassette containing the KASH sequence fused to GFP, enabling EGFP expression in the nuclear envelope ^59^ and allowing visualization of the viral infusion site (Fig 6B).

Animals with the CRISPR-mediated nonsense mutation of *Slc32a1* showed no changes in face interaction or aggression escalation behaviors (Fig 6C–E, FigS6). However, isolation-induced attack was significantly reduced in these mice (Fig 6F) regardless of sex (Fig S6 D, H). This robust reduction in attack, with no impact on any investigative behaviors, reveals that release of GABA from mPFC Tac2^+^ neurons controls the release of aggression, whereas NkB signaling from the same population of neurons controls the escalation of aggression (Fig 6G).

## DISCUSSION

Neuropeptides are thought to play a privileged in the control of internal states ^1^. Recent findings have begun to reveal the mechanisms by which neuropeptides exert precise and coordinated control over motivated behavior. In particular, the subcortical action of distinct neuropeptides ^6,7^ suggest that neuropeptides wield influence over behavior through their ability to work antagonistically. In contrast, little is known about the mechanism by which neuropeptides control a single behavioral state comprised multiple phases. Here, we identify that release of a stimulatory neuropeptide and the co-release of GABA enable the coordinated control of sequential, mutually exclusive actions that tile a behavioral state, despite being opposing in their sign. Our work contributes a new mechanism by which a single population of neurons can control distinct behaviors through both release of their slow-acting, long-range neuropeptides and their co-release of fast-acting, locally acting classical neurotransmitters.

### Social isolation induces an aggressive state comprised of mutually exclusive actions

Prior work from our group and others has established that prolonged social isolation induces aggression ^9,60^, ^61 21^. Here, we identified isolation-induced increases in aggression in both male and female mice. Our behavior forward approach further revealed that the state of aggression induced by isolation is comprised of a series of behaviors that sequentially tile this state. Indeed, we found that isolation increased attack behavior, as well as a number of social investigatory behaviors, including those directed towards the face, body and anogenital region. Importantly, this isolation-induced increase in investigatory behavior was most pronounced during the period of social exchange preceding aggression, with investigation inhibited once animals shifted to attack behavior. The effect of isolation to promote increases in social investigation in the escalation to attack, as well as the promotion of attack behavior, suggests that these two classes of behavior tile the trajectory of an aggressive encounter, consistent with the idea that once animals enter the attack phase, other behaviors are shut down.

Interestingly, face interaction, or face-to-face contact – sometimes referred to as face “threat” ^62^ or a “hard look” ^63^, has been implicated in threatening encounters. However, we found that although isolation increased face interaction, mPFC Tac2^+^ neuron activity showed no tuning to this behavior. This suggests that face interaction may be an impact of social isolation that is distinct from the effect of isolation to induce aggressive escalation or attack. This is consistent with all of our loss-of-function perturbations, where face interaction remained unchanged. This suggests that face interaction may be controlled by a distinct signaling system operating in parallel to Tac2 and sensitive to isolation. These findings suggest that while isolation induces a state of aggression, this state is comprised of a series of mutually exclusive behaviors which must be executed in a coordinated fashion.

### Neuropeptide signaling beyond subcortical regions

We previously found that Tac2/NkB action within sub-cortical structures controls the state induced by isolation and its impact to promote deleterious behaviors ^9^. Our cell-type specific neural recordings and targeted loss-of-function findings extend the role of Tac2/NkB beyond its function across subcortical regions, revealing a novel role for Tac2+ cells in the cortex in the control of isolation-induced aggression.

Classically, the idea that neuromodulators play a specialized role in the control of internal states has largely focused on their signaling properties in subcortical brain regions ^1,8,64,65^. In contrast, the role of neuropeptides and their signaling in the cortex has been relatively understudied. Recent work has begun to reveal a role for neuromodulatory signaling in the mPFC in the control of fear ^66^, suggesting that neuromodulatory action within the cortex may be critical for the top-down control over internal states. Here, we reveal that Tac2^+^ neuronal activity, signaling, and co-release of GABA from these cells, is critical for exerting control over distinct isolation-induced aggression. To our knowledge, these data represent the first discovery of Tac2^+^ neuron function in the cortex. Our results are consistent with a broader role for PFC in selecting behavioral responses based on internal and external information from converging inputs ^12^ and its control over behavioral transitions ^66,67^.

### Tac2^+^ neurons “release the brakes” on aggression

Our fiber photometry recordings demonstrate that Tac2^+^ neurons are active during isolation-induced aggression. Alternatively, prior work has identified a role for mPFC pyramidal neurons (PNs) in inhibiting aggression ^21,26,68,69^, suggesting that PNs may be tonically active to limit aggression. In other words, mPFC PNs are thought to put the “brakes” on aggression ^70, Li, 2025 #4^. Given that we identified Tac2^+^ neurons in the cortex to be GABAergic, this suggests the exciting possibility that Tac2^+^ neurons may control aggression via their ability to locally inhibit PNs, consistent with our cFos studies, where we found that isolation increased Tac2^+^ activity. Thus, isolation could drive Tac2^+^ neuron activity, which could then inhibit PNs to “release the brakes” over aggression. This idea is further supported by our anterograde viral tracing data revealing that Tac2+ mPFC cells have axonal arbors that span the entire mPFC. Thus, Tac2^+^ neurons sit in an ideal anatomical position – and contain the appropriate genetic machinery – to enable the inhibition of long-range projection neurons whose activity normally inhibits aggression. Alternatively, Tac2+ mPFC cells could exert control over an aggressive state using a multi-node circuit mechanism which inhibits PNs via NkB-mediated activation of a separate class of GABAergic interneurons, which in turn inhibit PNs. Precedence for the existence of such incoherent feed forward loops has been identified by others ^71^.

### Distinct, co-released molecules for dissociable but integrated actions

Using three loss-of function approaches, we identify a role for Tac2^+^ neuron activity, Tac2/NkB signaling, and co-release of GABA in the control of isolation-induced aggression. Surprisingly, our shRNA-mediated knockdown of Tac2 reveals that Tac2/NkB controls the aggressive escalatory behaviors that lead up to attack, while our CRISPR-mediated mutagenesis of the gene encoding vGAT reveals that co-release of GABA controls the release of attack itself. These findings reveal that a single population of neurons is able to control the aggressive state induced by isolation via the release of two opposing signaling molecules. Importantly, they suggest that the long-acting, far-reaching, and potentially cumulative impact of neuropeptide release across a long period of time (e.g., the window of isolation) is sufficient to put an animal in a heightened state of arousal, characterized by escalatory behaviors. In contrast, the fast acting, quick and localized effect of GABA release is well-suited for the tight, time-locked control over attack behavior, including the ability to inhibit PNs that normally exert brakes on aggression. Collectively, this pair of results are the first to demonstrate that a single population of neurons, co-releasing opposing signaling molecules, controls mutually exclusive behaviors that tile a single behavior state, via the utilization of distinct signaling properties of each molecule.

Interestingly, DREADD-mediated inhibition of Tac2^+^ neurons reduces aggression, but has no effect on investigation, thereby phenocopying the effects of disrupting GABA signaling from Tac2^+^ neurons, but not the effects of *Tac2* knockdown within the same cells. We reason that this may be due to the nature of our chemogenetic LOF approach, as there is evidence that hM4Di DREADDs not only hyperpolarize neurons via G-protein-gated inwardly rectifying potassium (GIRK) channels but are also able to inhibit neurotransmitter release through the βγ subunit’s interaction with the SNARE protein, SNAP-25 ^72,73^, potentially explaining the effects of this approach to disrupt GABAergic signaling but not neuropeptide signaling. While there is evidence that SNAP-25 is part of neuropeptidergic dense core vesicle fusion and release^74,75^, it remains understudied, making it difficult to assess whether hM4Di-mediated neuronal inhibition would impact neuropeptide release. Additional LOF approaches are warranted to further explore whether mPFC Tac2^+^ neuron activity is required for isolation-induced increases in investigatory behaviors.

### Implications for the treatment of pathological aggression

Aggression is a social behavior, critical for survival. However, when elicited in excess or as a result of stress, aggression can become pathological ^70^. As with other forms of pathological behavior, the mPFC is thought to offer promise as a region capable of exerting top-down control over aggression ^70,76,77^. Here, we identify the neuropeptide NkB as a critical player in the circuitry of isolation induced aggression – not just subcortically, but in the cortex as well. We find that Tac2^+^ cells in the mPFC enable a delicate balance between the behaviors displayed during an aggressive encounter in response to social isolation. These findings suggest a target region, and target molecule, for the control of isolation-induced aggression, and reveal novel motifs for the coordinated control of sequential behaviors.

## METHODS

### Animals

Wild-type (WT) C57BL/6N male and female mice and BALB/C intruders (male and female) were obtained from Charles River (7–10 weeks of age). For Cre-dependent viral manipulation experiments, we used the Tac2-IRES-Cre strain ^78^ and the Tacr3^iCre:GFP^ (Jackson labs) which were backcrossed to C57BL/6N at the University of Utah animal facility. Mice were housed and maintained on a reverse 12-hr light-dark cycle with food and *water ad libitium*. All behavior testing was performed during the dark cycle. Experimental manipulations and animal care were performed following the National Institutes of Health Guide for Care and Use of Laboratory Animals and approved by the University of Utah Institutional Animal Care and Use Committee.

### Resident Intruder assay

8–10-week-old C57Bl6/N mice (Charles River, strain code: 027) were either single housed or remained group housed in cages of four for two weeks. For the Resident Intruder (RI) assay, mice were transferred to the behavioral testing room and allowed to acclimate in the dark for approximately 30 minutes before the start of testing. Isolated mice remained in their home cage for the duration of testing. To begin animals were placed under an infrared Basler camera for and video recorded for three minutes as a baseline, then an age- and sex- matched Balb/c (Charles River, strain code: 028) intruder was then placed in the cage for 10 minutes as the test phase. For group housed animals, untested cage mates were removed from the home cage and placed in a clean holding cage, then once a given animal had been tested it was moved to a clean holding cage with its tested cage mates. Following the completion of testing for all mice in each cage, the mice were returned to their home cage. The apparatus was cleaned with 70% ethanol between all tests to reduce olfactory cues.

#### Behavior scoring.

All behavior (attack, mounting, face investigation, body investigation, anogenital investigation) were scoring by a trained, blinded observer using Noldus Observer, data was then exported to Excel (Microsoft).

#### Transition matrices.

The transition matrices were generated by calculating the probably of a mouse transition from one behavior to the next within a one second window of time, see^44^.

### RNAscope (fluorescent in situ hybridization)

For cFos RNAscope^™^ experiments mice were anesthetized using isoflurane (4–5%), animals were rapidly decapitated, and brains extracted 30 minutes from the start of the test phase of the RI assay. Brains were placed into cold 2-methylbutane (Sigma-Aldrich, M32631–4L) for approximately ten seconds or until frozen, then wrapped in foil and stored at −80°C for cryo-Sectioning. For RNAscope protocol see below section.

Brains were rapidly extracted and flash frozen in 2-methylbutane (Sigma-Aldrich, catalog #: M32631–4L) and stored at −80C for up to 4 months. Sections were taken at 20μm thick and mounted directly onto slides (SuperFrost PLUS Adhesion slide, Electron Microscopy Sciences, catalog #: 71869–11) in the cryostat. Roughly 4–5 sections from two animals, one per housing condition were placed on each slide. Slides were stored in airtight containers at −80°C for up to four months before the RNAscope^™^ assay was performed. The RNAscope^™^ assay was performed according to the manufacturer’s instructions (Advanced Cell Diagnostics, see Wang et al 2012). The following probes (ACDBio) were used: *Tac2*, *Pvalb*, *Som*, *cFos, Tacr3*, *Vip*, *Crh, Slc17a7, Slc32a1*. For the experiments using *Gad1* and *Gad2* probes, a 50:50 mixture of each probe was used and is indicated by “*Gad1/2*”. The slides were then imaged on a Keyence BX-X800 within a week of the assay.

### Single nucleus RNA sequencing

The mPFC (prelimbic and infralimbic) was dissected form three group housed and three isolated C57Bl/6N males (strain code: 027, isolated for three weeks) in ice cold RNase free 1X PBS containing 15um actinomyocin-D ^79^ (Sigma-Aldrich, catalog #A1410–10MG). Nuclei were isolated using manual homogenization and the Nuclei PURE Prep kit (Millipore Sigma, catalog # NUC201–1KT) with 15 μM actinomycin-D added to the lysis buffer. Nuclei were resuspended in a resuspension buffer containing 0.5% Ultrapure BSA (Fisher Scientific #AM2616) to prevent clumping of nuclei and 0.2 U/μl RNase inhibitor (New England Biolabs #M0314S) to prevent RNA degradation ^80^. Single-nuclei barcoded cDNA libraries were generated by the University of Utah High Throughput Sequencing using the 10x Genomics user guide “Chromium Next GEM Single Cell 3’ Reagent Kits v3.1”. Libraries were sequenced on the Illumina NovaSeq 6000. Fastq files were aligned to the GRCm39 reference genome using 10X Cell Ranger (v4.0.8). Filtered gene expression matrices were then imported into R (v4.3.0) and nuclei with fewer than 800 features, greater than 6000 features, or greater than 5% mitochondrial reads were removed to eliminate low quality nuclei and multiplets. Seurat (v4.2.1)^81^ was used to calculate nearest neighbors at 0.8 resolution from the first 30 principal components and cluster nuclei using UMAP dimensionality reduction. Then clusters were assigned to known cell types based on expression of known cell type specific marker genes ^82,83^.

### Surgical procedures

Mice were anesthetized in an induction chamber using 4–5% isoflurane and anesthesia was maintained at 1–2% for the duration of the procedure while mice were mounted on the nose cone of the stereotaxic surgery rig (Kopf Instruments, California, USA). Once mounted in the nose cone, ophthalmic ointment (Soothe lubricant eye ointment, Bausch and Lomb) was applied to prevent eyes from during the procedure. Topical hair removal cream (Nair) was applied to the scalp to remove hair, following removal of cream and wiping with distilled water, the surgical area was cleaned 3x with povidone-iodine solution (Dynarex, catalog 1415) and isopropyl alcohol (Solimo). A subcutaneous injection of sustained release buprenorphine at a dose of 3.25 mg/kg animal weight (Ethiqa XR 1.3mg/l mL, Fidelis Animal Health) was given before incisions to provide analgesia. A small incision was made along the scalp to expose the skull, and the skull was leveled between bregma and lambda as well as in the medial/lateral direction. Holes were then drilled at the coordinates for the viral infusion cites.

All infusion coordinates into the medial prefrontal cortex (infralimbic) were made in relation to brain in mm. For all experiments (DREADDs, Tac2 shRNA, visualization of Tac2^+^ neuron morphology, CRISPR mutagenesis) bilateral infusions were performed at AP: +2.14, ML: +/− 0.25, DV: −2.79. Virus was backfilled into pulled fine glass capillaries (~ 50 μm diameter tip; World Precision Instruments, item no. 504949) filled with mineral oil (sigma Aldrich, M5904, CAS 8042–47-5) and 140nL virus (each side) was infused at a rate of 80nL/min using a nanoliter injector (Nanoliter 2000, World Precision Instruments) controlled by an ultra-micro-syringe pump (Micro4, World Precision Instruments). Capillaries remained in place for 5 minutes following viral infusion to allow for full diffusion of virus and to reduce backflow up the infusion tract. The scalp was drawn together and sealed with GLUture (World Precision Instruments, catalog 503763). All infusion sites were verified using epifluorescent microscopy.

### Characterization of Dendritic Arbor of mPFC Tac2 neurons

Male and female Tac2-IRES-Cre mice 8–10 weeks were single housed and had surgeries performed to infuse a Cre-dependent adeno-associated virus (AAV) expressing membrane-GFP and synaptophysin fused to mRuby (AAV2-hSynb-FLEx-mGFP-2A-Synaptophysin-mRuby) infused into the mPFC (Addgene #: 71760-AAV2, titer: 2.7 × 10^12^ GC/mL, lot: v138755). Virus was allowed to express for approximately four weeks, then animals anesthetized with isoflurane (4–5%) and rapidly decapitated. Brains were post fixed in 4% paraformaldehyde (CAS 30525–89-4, Sigma Aldrich, catalog #: 158127–500G) overnight at 4°C, then transferred to 15% sucrose overnight at 4°C, followed by 30% sucrose overnight at 4°C. After the final sucrose transfer brains were embedded in OCT (Tissue-Tek O.C.T Compound, Sakura Finetek USA Inc, catalog #: 4583) and stored at 80° until cryo-sectioning. Viral placement and expression were verified for all animals using epifluorescent microscopy.

### Fiber photometry

Group housed an isolated male and female Tac2-IRES-Cre mice (Jackson laboratories, strain#: 021878; ~9–11 weeks old) were infused with 300µL of a Cre-dependent GCaMP6f (AAV1-Syn-Flex-GCaMP6f-WPRE-SV40; Addgene #10083; coordinates: AP +2.13, ML: +0.3, DV-2.78) into the mPFC and a fiber optic cannula (Doric, MFC_400/430–0.66_3.0mm_ZF2.5_FL) was implanted along the viral tract at 0.1mm above the DV coordinate of the viral infusion site. After implantation the skull was covered in metabond (Parkell C&B-Metabond Quick Luting Cement base and Radiopaque L-Powder S398) and PEMA temporary resin acrylic (Bosworth Trim II) to secure the implant.

#### Behavior:

Two days before behavior, mice were habituated to the cable, by connecting and moving with cable attached to the optic cannula in the Resident Intruder chamber for 10 min. On test day, behavior was recorded while a subject freely located their home cage for 5min, then an age and sex-matched Balb/c intruder was introduced and behavior was recorded for 15 minutes. The RI test was conducted two times using a novel intruder. Five different behaviors (face threat, body, rear investigation, attack, and mounting) were scored by a blinded experimenter manually using Noldus Observer. Mounting and attack were summed to make the category of “attack”.

#### Fiber photometry recording data analysis:

The fiber photometry recordings give two output signals, one at 415 nm (isosbestic Ca^2+^-independent signal for motion correction) and the other at 470 nm (Ca^2+^-dependent signal). The data analyses were done as previously described^84–86^. First, the 415 nm signal was aligned to the 470 nm signal using a least-squares linear fit. Then, motion-corrected 470 nm signal was obtained by (470 nm signal - fitted 415 nm signal)/fitted 415 nm signal. The motion-corrected 470 nm signal is then scaled from zero to one, using the pre-intruder activity (mean activity during 35 to 5s before intruder introduction) as a baseline and z-scored. dF/F during the five seconds before and after the behavior onset was z-scored by subtracting the mean of the baseline dF/F and then dividing by the standard deviation of the dF/F signal. Trials were excluded if there were indications that the fiber connection was poor as indicated by a Ca^2+^ signal that failed to increase 2 standard deviations above the mean dF/F (baseline window: 30s, −35s to −5s before intruder in) within a ten second window beginning when the intruder was introduced. A 0.5s Gaussian smoothing was applied only to the figures.

In animals that showed at least one bout of attack behavior (N=3 animals, 16 bouts across all animals), Tac2^+^ neuron activity around the onset of aggression behavior was examined using a time window of −2 seconds (before) to + 4 seconds (after) the behavior onset. The signal (dF/F) around aggression was compared to shuffled data, generated by circularly shuffling the real data (whole recording ~20 min) 1000 times, creating 100 datasets. Each shuffled dataset contains all data points coming from the original data, but the temporal relationship is disrupted. For analysis of face threat, body investigation and anogenital investigation only behavior bouts that were longer than 0.5s and separated by 5s from the previous behavior were used. If aggression occurred immediately after face threat or investigation, the baseline was determined using the window before the prosocial behavior. To calculate area under the curve for social/investigation behaviors, the area under the z-score curve during each behavior was calculated from 0s to 4s. For attack, the area under the z-score curve during from the time window of 2s post behavior onset to 4s post behavior onset.

### DREADD mediated inhibition

Male and female Tac2-IRES-Cre mice were single housed starting at 8–10 weeks of age for three weeks to induce social isolation, then the Resident Intruder assay was used to screen for attack. The pre-screening test was a shortened RI paradigm with a two-minute baseline and 8-minute test phase, mice showing any aggressive behavior (attack, mounting) were considered “aggressive.” Animals that showed at least one attack bout were then used for neural manipulation experiments. To manipulate the activity of *Tac2* neurons, a viral-mediated Cre-dependent DREADDs construct was infused into the mPFC of Tac2-IRES-Cre mice (Jackson laboratories, strain#: 021878) via stereotaxic surgery. Mice were anesthetized with 4% isoflurane (MWI Animal Health, VetOne, MWI #: 502017) and mounted on a stereotaxic frame with a nose cone for anesthesia. In Tac2-IRES-Cre mice, 140nL of a Cre-dependent inhibitory DREADDs virus (AAV2-hSyn-DIO-hM4Di-mCherry, Addgene # 44362, titer 3.3×10^13^ GC/mL, lot v138755) was infused bilaterally into the of aggressive Tac2-IRES-cre mice. Following surgeries mice remained single housed for three to four weeks before Resident intruder.

For behavioral testing, Tac2-IRES-Cre mice received 0.1 mg/kg body weight Deschloroclozapine (DCZ, CAS: 9177–07-7, MedChemExpress, catalog #: HY-42110) or vehicle (DMSO/saline) via intraperitoneal injection 10 minutes before the start of the baseline phase of the RI assay (see above for detailed information). Mice were randomly assigned to receive a given drug on a given day so for each round of behavior testing there were approximately even numbers of mice receiving DCZ on the first day and vehicle on the first day. After the first test mice were administered the other solution, i.e. if the animal received DCZ on the first test day, they then received the vehicle solution on the second test day 48hrs after the first test day. Video was recorded during baseline and test phases in infrared light using a high-speed Basler camera. Following behavior, brains were taken and post-fixed in 4% Paraformaldehyde (CAS 30525–89-4, Sigma Aldrich, catalog #: 158127–500G) overnight at 4°C, then transferred to 15% sucrose overnight at 4C, followed by 30% sucrose overnight at 4°C. After the final sucrose transfer brains were embedded in OCT (Tissue-Tek O.C.T Compound, Sakura Finetek USA Inc, catalog #: 4583) and stored at −80° until cryo-sectioning. Viral placement and expression were verified for all animals using epifluorescent microscopy.

To determine whether DCZ had off-target effects on mouse behavior in the resident intruder assay, male and female WT littermates of animals from the Tac2-IRES-Cre and colonies were injected with two of four doses of DCZ (0.1mg/kg, 0.25mg/kg, 0.5 mg/kg or 1.0 mg/kg body weight) to account for variability in animal behavior. The days on which animals received vehicle or DCZ was counterbalanced. The behavioral testing on RI occurred just as in the DREADDs experiments, with the exception that mice were tested on the second dose of DCZ approximately one week after the first two test days.

Statistical analysis on the duration of behaviors on vehicle and DCZ was performed using GraphPad PRSIM. Only animals that engaged in aggression (mounting, attack) on at least one of the test days and had virus on at least one side of mPFC were included in the analyses. Normality tests and QQ plots were performed, and a Wilcoxon test was performed if data was not normally distributed, if data was normally distributed then a paired t-test was performed.

### Immunohistochemistry

For cFos immunohistochemistry experiments, mice underwent transcardial perfusions with 0.9% saline (MWI Animal Health, catalog # 033500) followed by 4% paraformaldehyde (PFA; Paraformaldehyde powder, CAS 30525–89-4, Sigma Aldrich, catalog #: 158127–500G) 90 minutes after the middle of the test phase of the Resident Intruder assay and post-fixed in 4% (PFA) overnight at 4°C. Brains were then transferred to 15% sucrose solution (Sigma-Aldrich, S0389–5KG, CAS 57–50-1) at 4°C overnight. Then brains were then transferred to 30% sucrose solution overnight at 4°. Brains were then sectioned at 40um into 1X PBS (Gibco, ref 10010–023) and stored at 4C for up to a week before immunohistochemistry was performed. Sections were incubated in 10% Normal Donkey Serum (Sigma-Aldrich, catalog #: D9663–10mL) + 0.3% Triton X-100 (Sigma, X-100) in 1X PBS (Gibco, ref 10010–023) for one hour at room temperature. Then sections were incubated in the primary antibody (rabbit anti-cFos, Abcam 190289) at 4°C for 72 hrs. Following this incubation, sections were rinsed in 1X PBS (Gibco, ref 10010–023) three times then incubated in secondary antibody (Donkey anti-rabbit Alexa Fluor-488, 1:250 dilution in blocking buffer, catalog A-21206, ThermoFisher) for 12 hours. After this period, sections were rinsed three times in 1X PBS (Gibco, ref 10010–023), then mounted onto slides (SuperFrost PLUS Adhesion slide, Electron Microscopy Sciences, catalog #: 71869–11) and counterstained with DAPI mounting media (DAPI Fluoromount-G Mounting Medium, Liquid, Southern Biotech, catalog # 0100–20). Slides were imaged with epifluorescent microscope (Keyence BX-Z800) by a blinded experimenter within seven days of mounting. The number of cFos^+^ cells and total number of cells (DAPI) were quantified on sections with viral expression in mPFC (FIJI). GraphPad PRISM was used to perform the nested t-tests for the number cFos^+^ cells out the total number of cells on tissue from each drug condition.

### Tac2 shRNA experiments

Male and female 8-week-old C57Bl6/N mice (Charles River, strain code: 027) were single housed for two-three weeks, then pre-screened for aggression. Aggressive mice were randomly assigned two approximately equal sized groups and infused with either the Tac2 shRNA virus or a luciferase control virus (plasmids courtesy of Tomomi Karigo and David Anderson; see Zelikowsky et al 2018) into the mPFC. After three weeks of expression and recovery, animals were tested on the Resident Intruder assay. Statistical analysis for the duration for behaviors between shLuciferase vs shTac2 animals was performed using GraphPad PRISM on animals expressing virus in at least one side of mPFC. Normality tests were performed for the data in each behavior category and unpaired t-tests were performed (normally distributed: unpaired t-test, not normally distributed: Mann-Whitey test).

Following the completion of behavioral testing, brains were flash frozen within three days and sectioned in preparation for RNAscope^™^. For the RNAscope^™^, each slide contained two mice, one from each viral condition, and the Tac2 probe was used to examine for Tac2 knockdown. Slides were imaged using the Keyence BX-Z800. Experimenters were blinded to both the sex and viral condition of animals during RNAscope^™^, imaging, behavior scoring and cell counting. Knockdown of Tac2 was verified by taking automated counts of DAPI and manual counts of Tac2 (quantified by fluorescence in Tac2 channel that colocalized with DAPI signal) in FIJI. GraphPad PRIM was used for nested t-test of cell count data. Viral placement and expression were verified for all animals using epifluorescent microscopy.

### CRISPR mutagenesis experiments

Male and female 8 – 10-week-old Tac2-IRES-Cre mice received bilateral infusions of a 1 EGFP-KASH: 9 parts saCas9 mixture of either AAV1-FLEX-saCas9-U6-sgSlc32a1 and AAV1-EGFP-KASH or AAV1-FLEX-saCas9-U6-Rosa26 and AAV1-EGFP-KASH (Gift from Zweifel lab). Following surgery mice were single housed for four weeks to allow for viral expression. After this period, mice were tested on the Resident Intruder assay as described above and blinded experimenters scored behavior (Noldus Observer) to quantify to duration of aggression, face investigation, anogenital investigation and body investigation. Data was then analyzed in GraphPad PRISM on animals expressing virus in at least one side of mPFC, normality tests were performed and for all data that was not normally distributed, Mann-Whitney tests were performed and unpaired t-tests were performed on normally distributed data.

Brains were extracted within a week of behavioral testing, post-fixed in 4% paraformaldehyde at 4°C overnight, then 15% sucrose and 30% overnight before embedding in OCT (Tissue-Tek O.C.T Compound, Sakura Finetek USA Inc, catalog #: 4583) and storage at −80°C. Cryo-sectioning was performed and the presence and location of virus in the brain was recorded using epifluorescent microscopy by a blinded experimenter.

## Supplementary Material

Supplement 1
