# Supplementary material for: Co-release of opposing signaling molecules controls the escalation and release of aggression
Source: bioRxiv. 2025 Jun 8:2025.03.13.643119. Preprint. [Version 2] doi: 10.1101/2025.03.13.643119 (PMC12157652; doi:10.1101/2025.03.13.643119)
Supplement: Supplement 1 [file media-1.pdf]

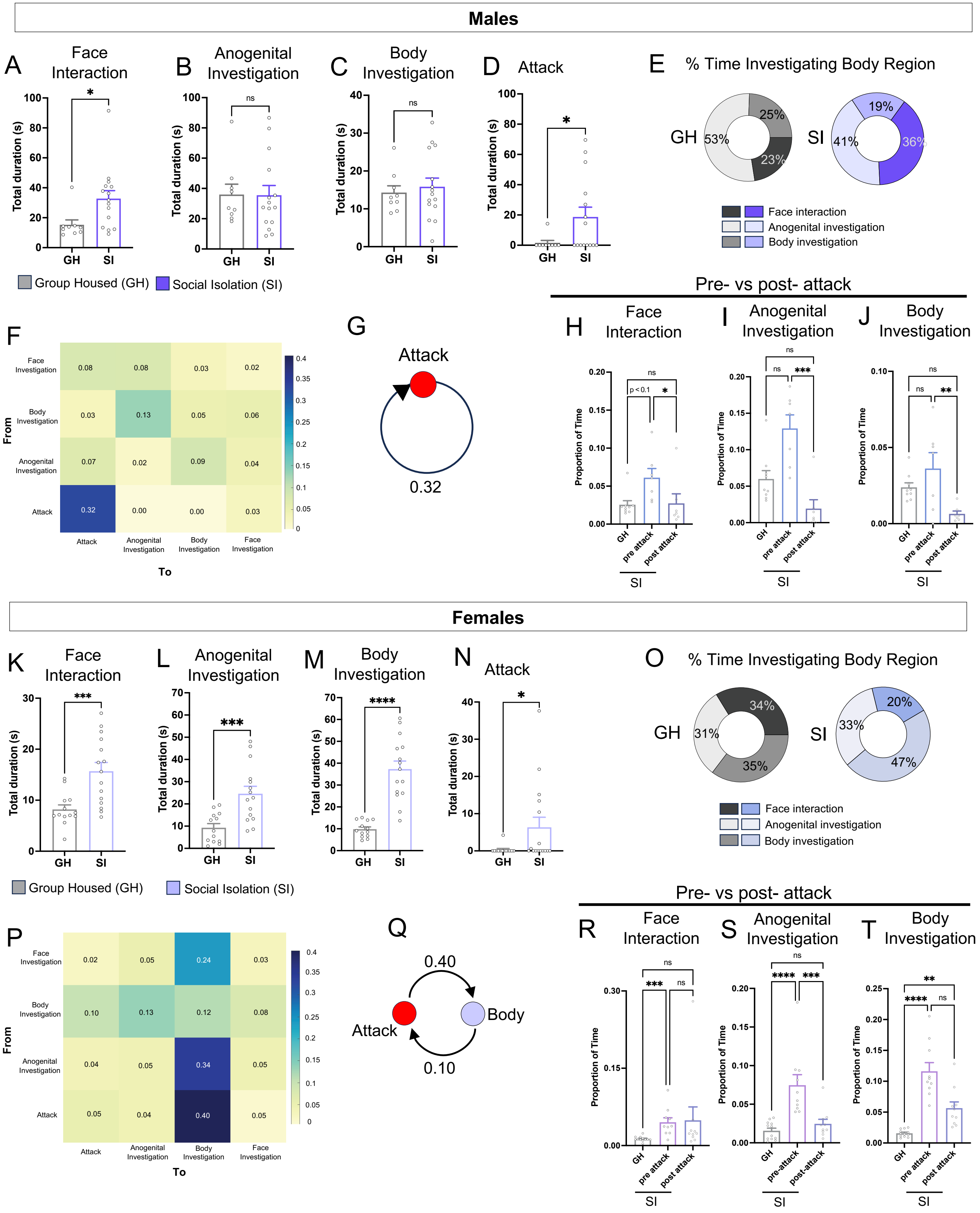

Males
A
Face Interaction
Total duration (s)
GH
SI
*
B
Anogenital Investigation
ns
C
Body Investigation
D
Attack
E
% Time Investigating Body Region
GH
53%
25%
23%
SI
41%
19%
36%
Face interaction
Anogenital investigation
Body investigation
Group Housed (GH)
Social Isolation (SI)
F
Face Investigation
Body Investigation
Anogenital Investigation
Attack
From
To
0.08
0.08
0.03
0.02
0.03
0.13
0.05
0.06
0.07
0.02
0.09
0.04
0.32
0.00
0.00
0.03
G
Attack
0.32
Pre- vs post- attack
H
Face Interaction
I
Anogenital Investigation
J
Body Investigation
Proportion of Time
pre attack
post attack
p < 0.1
***
**
Females
K
Face Interaction
***
L
Anogenital Investigation
M
Body Investigation
****
N
Attack
O
% Time Investigating Body Region
GH
34%
31%
35%
SI
20%
33%
47%
P
0.02
0.05
0.24
0.03
0.10
0.13
0.12
0.08
0.04
0.05
0.34
0.05
0.05
0.04
0.40
0.05
Q
0.40
Attack
Body
0.10
R
Face Interaction
S
Anogenital Investigation
T
Body Investigation
pre-attack
post-attack

Supplemental Figure 1. Prolonged social isolation induces aggression in males and females; corresponds to Fig 1.

A-J Males.
A. Total duration (s) of face interaction towards intruder in group housed (GH) and isolated (SI) males; Mann-Whitney test, $p < 0.05$
B. Total duration (s) of anogenital investigation towards intruder, Mann-Whitney test $p > 0.05$.
C. Total duration (s) of body investigation towards intruder, unpaired t test $p > 0.05$.
D. Total duration (s) of aggression towards intruder in group housed (GH) and isolated (SI) males; Mann-Whitney test, $p < 0.05$.
E. Pie charts showing percent of total investigation directed towards body region (face, anogenital, or body).
F. Transition matrix showing probability of aggressive isolated males transitioning from one behavior to another.
G. Diagram showing isolated males attack cycle.
H. Proportion of time group housed (GH) males and isolated males (pre and post attack) engage in face interaction; Kruskal-Wallis test $p < 0.05$, Dunn's multiple comparions test: GH vs pre attack $p < 0.1$, GH vs post attack $p > 0.05$, pre attack vs post attack $p < 0.05$.
I. Proportion of time engaged in anogenital investigation; GH: group housed, pre-attack: isolated male before first attack, post attack: isolated males after first attack; Kruskall-Wallis test, $p \leq 0.01$, Dunn's multiple comparison's test: GH vs pre-attack $p > 0.05$, GH vs post attack $p > 0.05$, pre attack vs post attack $p < 0.001$.
J. Proportion of time engaged in body investigation; GH: group housed male, pre-attack: isolated male before first attack, post attack: isolated males after first attack; One-way ANOVA, $p < 0.01$, Tukey's multiple comparison's test: GH vs pre-attack $p > 0.05$, GH vs post attack $p > 0.05$, pre attack vs post attack $p < 0.01$.
K-T. Females.
K. Total duration (s) of face interaction with intruder in group housed (GH) and isolated (SI) females, unpaired t-test $p = 0.001$.
L. Total duration (s) of anogenital investigation towards intruder in group housed (GH) and isolated (SI) females, unpaired t-test $p < 0.001$.
M. Total duration (s) of body investigation towards intruder in group housed (GH) and isolated (SI) females, unpaired t-test, $p = 0.0001$.
N. Total duration (s) of aggression towards intruder in group housed (GH) and isolated (SI) females, Mann-Whitney test, $p < 0.05$.
O. Pie charts showing percent of total investigation directed towards body region (face, anogenital, or body).
P. Transition matrix showing probability of aggressive SI females transitioning from one behavior to another.
Q. Diagram showing isolated females transitioning between attack and body investigation.
R. Proportion of time group housed (GH) and isolated females (pre and post attack) engage in face threat; Kruskal-Wallis test $p < 0.01$, Dunn's multiple comparion's test.
S. Proportion of time engaged in anogenital investigation before and after the first instance of attack; GH: group housed female, pre-attack: isolated female before first attack, post attack: isolated females after first attack; One-way ANOVA, $p = 0.001$, Tukey's multiple comparison's test.

T. Proportion of time engaged in body investigation; GH: group housed female, pre-attack: isolated female before first attack, post attack: isolated females after first attack; Kruskal-Wallis test $p < 0.0001$, Dunn’s multiple comparison’s test.

*Bars represent mean ± SEM, dots within bars represent mean for each animal ns p > 0.05, * p < 0.05, ** p < 0.01, *** p < 0.001, **** p < 0.0001*

Tac2 cells are GABAergic

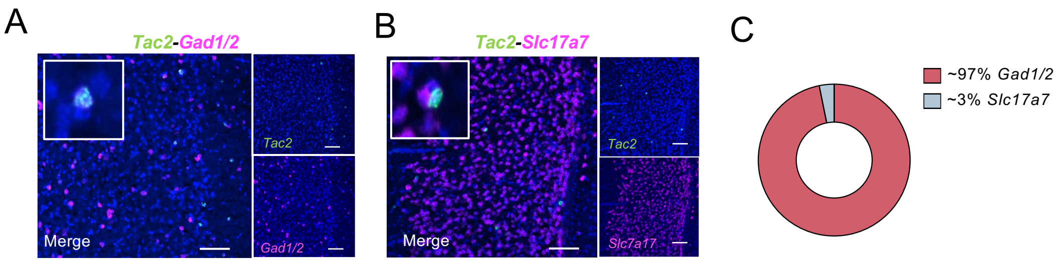

Tac2⁺ cells which co-express *Vip* are not preferentially active by isolation-induced aggression

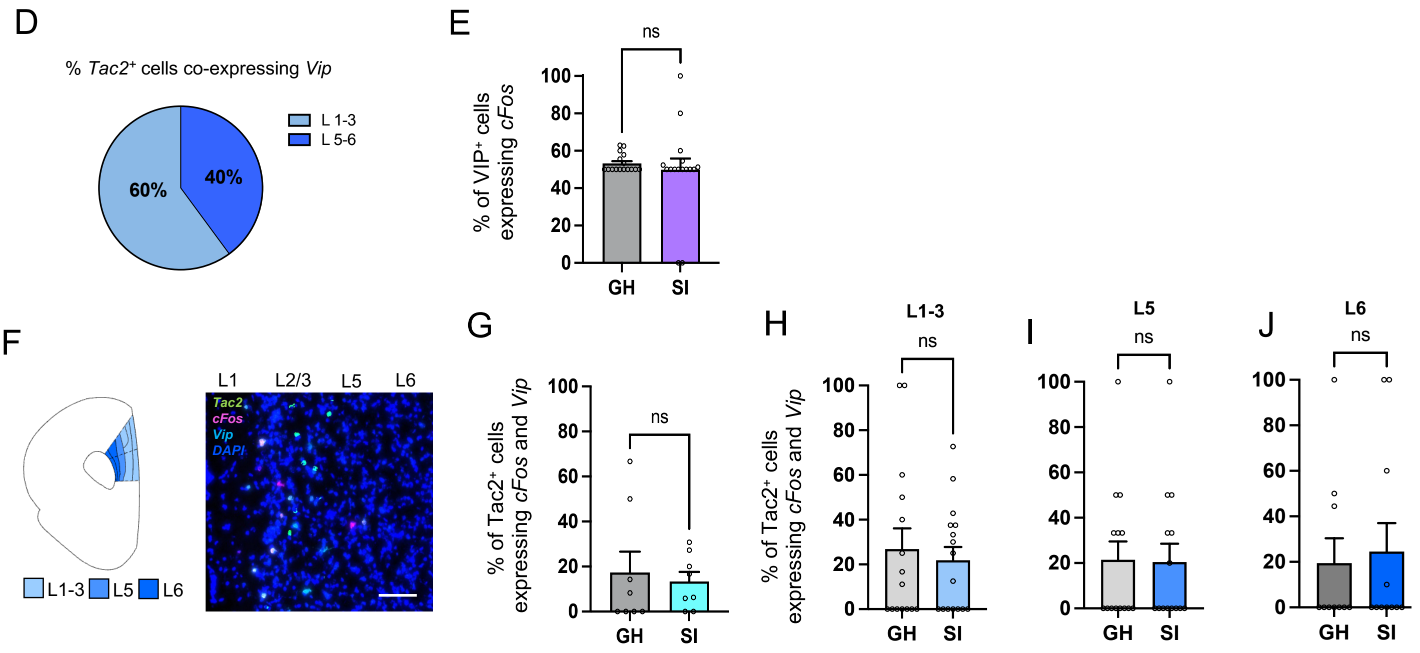

Tacr3/Nk3R cells are activated by resident intruder in a layer-specific manner

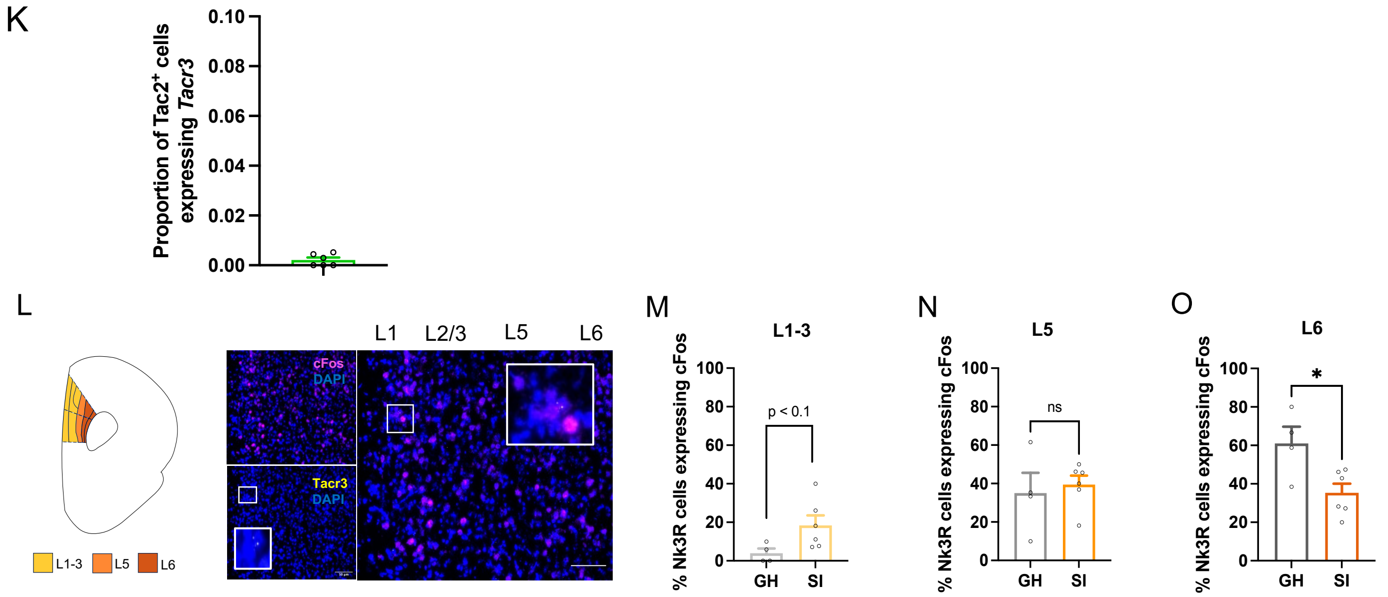

Supplemental Figure 2. Genetic characterization and activity profile of mPFC Tac2$^+$ neurons and Tacr3$^+$ neurons following the Resident Intruder assay; corresponds to Fig 2 and Fig 3

A. Representative image of in situ hybridization for *Tac2* and *Gad1/2* in infralimbic mPFC; scale bar represents 100μm.
B. Representative image of in situ hybridization for *Tac2* and *Slc17a7* in infralimbic mPFC; scale bar represents 100μm.
C. *Tac2* expression within *Gad1/2*$^+$ and *Slc17a7*$^+$ cells.
D. Percent of *Tac2*$^+$ cells in each layer that express *Vip*.
E. Bar graph showing percent of total *Vip* cells expressing *cFos* in mPFC, Mann-Whitney test, $p > 0.05$.
F. Representative image of in situ hybridization across cortical layers for *Tac2*, *cFos*, and *VIP* scale bar represents 100μm.
G-J. Females.
G. Percent of all Tac2$^+$ cells in mPFC expressing both *cFos* and *Vip*, Mann-Whitney test, $p > 0.05$.
H. Percent of Tac2$^+$ cells in layers 1-3 expressing both *cFos* and *Vip*, Mann-Whitney test, $p > 0.05$.
I. Percent of Tac2$^+$ cells in layer 5 expressing both *cFos* and *Vip*, Mann-Whitney test, $p > 0.05$.
J. Percent of Tac2$^+$ cells in layer 6 expressing both *cFos* and *Vip*, Mann-Whitney test, $p > 0.05$.
K. Percent of snRNAseq data showing proportion of *Tac2*-expressing cells that also express *Tacr3*.
L. Diagram of cortical layers in mPFC; representative image of FISH staining for *Tacr3* and *cFos* in mPFC, scale bar represents 100μm.
M. Percent of *Tacr3*$^+$ cells expressing *cFos* in layers 1-3 of mPFC, unpaired t-test $p < 0.1$.
N. Same as M, but in layer 5, unpaired t-test, $p > 0.05$.
O. Same as M-O, but in layer 6, unpaired t-test, $p < 0.05$

*Bars represent mean ± SEM, dots within bars represent counts from each section. ns $p > 0.05$, * $p < 0.05$*

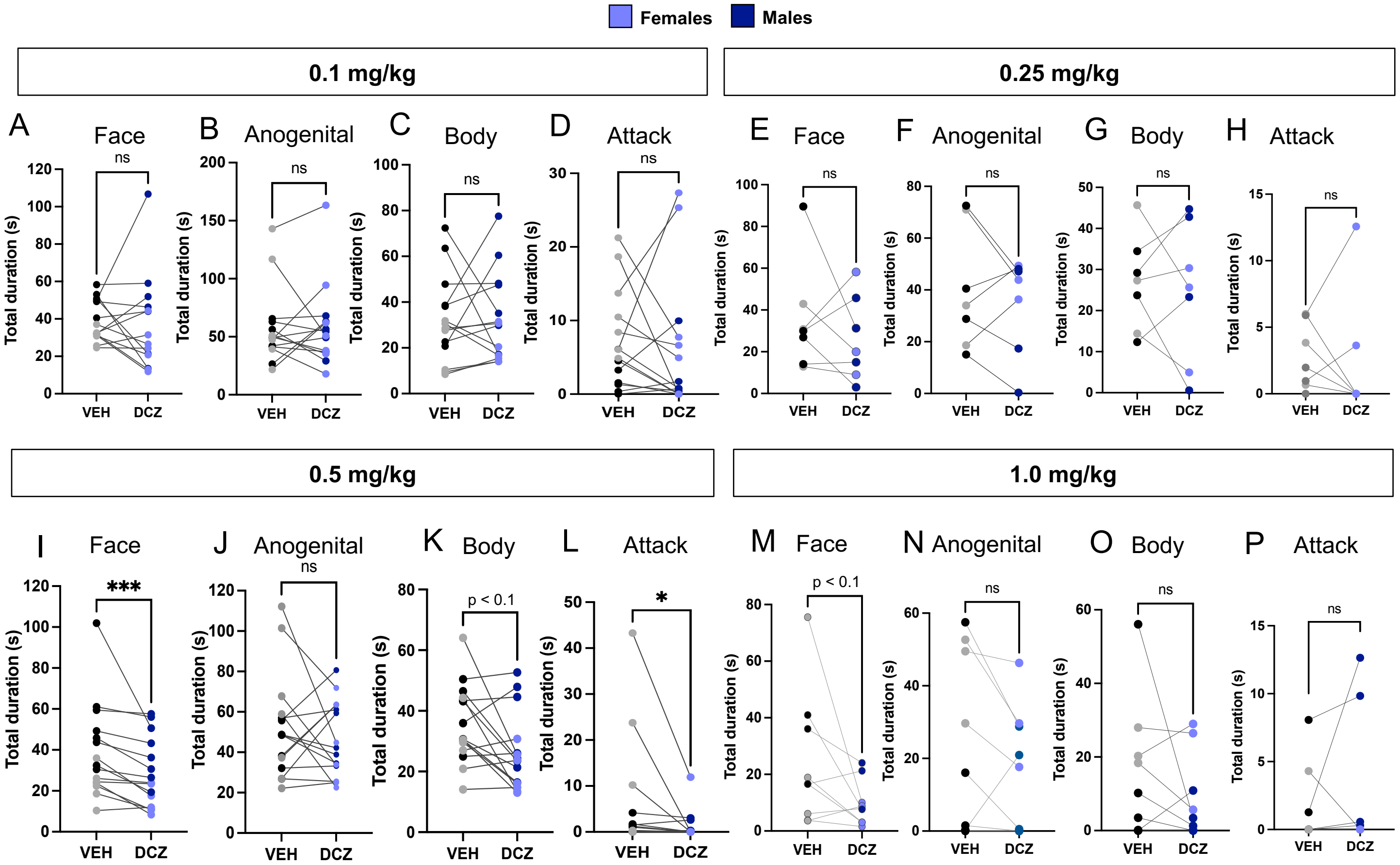

Females
Males
0.1 mg/kg
0.25 mg/kg
A Face
B Anogenital
C Body
D Attack
E Face
F Anogenital
G Body
H Attack
ns
Total duration (s)
VEH
DCZ
0.5 mg/kg
1.0 mg/kg
I Face
J Anogenital
K Body
L Attack
M Face
N Anogenital
O Body
P Attack
***
p < 0.1
*

Supplemental Figure 3. Dose-response relationship between DCZ and behavior in the Resident Intruder Assay; corresponds to Fig 4

A-F. 0.1 mg/kg DCZ.
A. Total duration of face threat when isolated animals received VEH and 0.1 mg/kg DCZ; Wilcoxon test, $p > 0.05$.
B. Same as A but showing total duration of anogenital investigation; Wilcoxon test, $p > 0.05$.
C. Same as A but showing total duration of body investigation; Wilcoxon test, $p > 0.05$.
D. Same as A, but showing total duration of aggression; Wilcoxon test, $p > 0.05$.
E. Workflow of cFos immunohistochemistry on animals expressing hM4D in mPFC Tac2 neurons.
F. Percent of cells expressing cFos in mice on vehicle (VEH) and Deschloroclozapine (DCZ), Nested t-test $p < 0.05$; bars represent mean ± SEM
G-J. 0.25 mg/kg DCZ.
G. Total duration of face threat when isolated animals received VEH and 0.25mg/kg DCZ; Wilcoxon test, $p > 0.05$.
H. Same as G but showing total duration of anogenital investigation; Wilcoxon test, $p > 0.05$.
I. Same as G, but total duration of body investigation; paired t-test, $p > 0.05$.
J. Same as G but showing total duration of aggression; Wilcoxon test $p > 0.05$. **K-N**. 0.5 mg/kg DCZ.
K. Total duration of face investigation when isolated animals received VEH or 0.5 mg/kg DCZ, N = 8 M, 7 F; Wilcoxon test $p < 0.001$.
L Same as K, but showing total duration of anogenital investigation, Wilcoxon test, $p > 0.05$.
M. Same as K, but showing total duration of body investigation, Wilcoxon test, $p < 0.1$.
N. Same as K, but showing total duration of aggression, Wilcoxon test, $p < 0.05$.
O-R. 1.0 mg/kg DCZ.
O. Total duration of face investigation when isolated animals received VEH and 1.0 mg/kg DCZ, N = 3 F, 6 M: Wilcoxon test, $p < 0.1$.
P. Same as O, but showing total duration of anogenital investigation, Wilcoxon test, $p > 0.05$.
Q. Same as O, but showing total duration of body investigation, Wilcoxon test, $p > 0.05$. **R**. Same as O, but showing total duration of aggression, Wilcoxon test, $p > 0.05$.

*Bars represent mean ± SEM, individual dots with connecting lines show mean duration of behavior for each animal on each drug. ns $p > 0.05$, *** $p < 0.001$.*

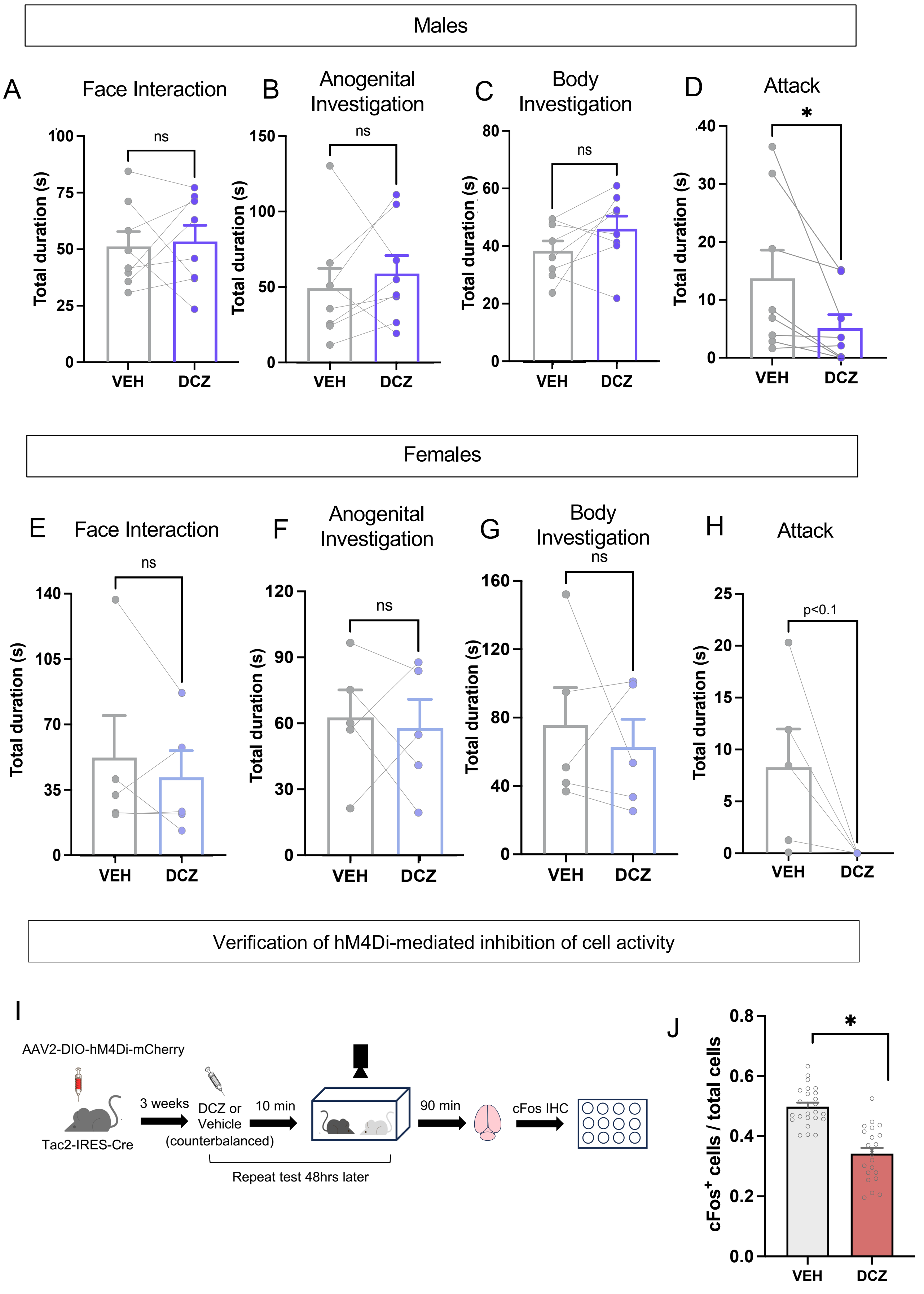
Males
A
Face Interaction
Total duration (s)
ns
VEH
DCZ
B
Anogenital Investigation
Total duration (s)
ns
VEH
DCZ
C
Body Investigation
Total duration (s)
ns
VEH
DCZ
D
Attack
Total duration (s)
*
VEH
DCZ
Females
E
Face Interaction
Total duration (s)
ns
VEH
DCZ
F
Anogenital Investigation
Total duration (s)
ns
VEH
DCZ
G
Body Investigation
Total duration (s)
ns
VEH
DCZ
H
Attack
Total duration (s)
p<0.1
VEH
DCZ
Verification of hM4Di-mediated inhibition of cell activity
I
AAV2-DIO-hM4Di-mCherry
Tac2-IRES-Cre
3 weeks
DCZ or Vehicle (counterbalanced)
10 min
90 min
cFos IHC
Repeat test 48hrs later
J
cFos+ cells / total cells
*
VEH
DCZ

Supplemental Figure 4. Sex-dependent effects of DREADD-mediated inhibition of mPFC Tac2 neurons; corresponds to Fig 4

A-E. Males.
A. Total duration of face threat isolated males expressing hM4D in mPFC Tac2 neurons, paired t-test, $p > 0.05$.
B. Same as a A, but showing total duration of anogenital investigation, Wilcoxon test, $p > 0.05$.
C. Same as A, but showing total duration of body investigation, paired t-test, $p > 0.05$.
D. Same as A, but showing total duration of aggression, Wilcoxon test, $p < 0.5$.
E-H. Females.
E. Total duration of face investigation in isolated females expressing hM4D in mPFC $Tac2^+$ neurons, Wilcoxon test, $p > 0.05$.
F. Same as E, but showing total duration of anogenital investigation, paired t-test, $p > 0.05$. **G**. Same as E, but showing the total duration of body investigation, paired t-test, $p > 0.05$. **H**. Same as E, but showing the total duration of aggression, Wilcoxon test, $p < 0.1$.
I. Workflow of immunohistochemistry for cFos following Resident Intruder assay to validate hM4D-mediated inhibition of mPFC $Tac2^+$ neurons.
J. Number of $cFos^+$ cells out of the total cells in hM4D-expressing mice given vehicle (VEH) and Deschloroclozapine (DCZ). Dots represent individual tissue sections; nested t-test, $p < 0.5$.

*Bars represent mean ± SEM, individual dots with connecting lines show mean duration of behavior for each animal on each drug. ns $p > 0.05$, * $p < 0.05$*

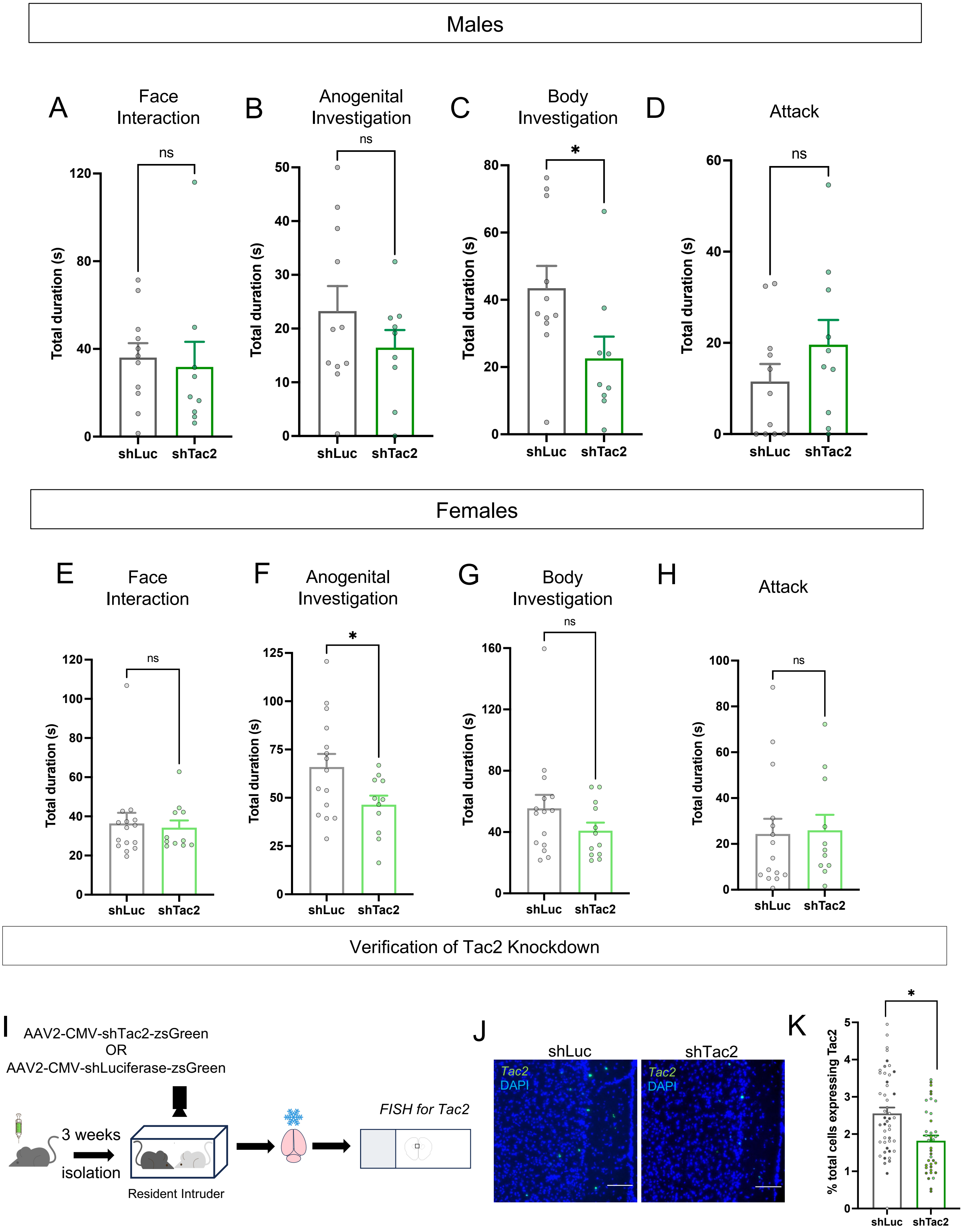
Males
A
Face Interaction
ns
Total duration (s)
120
80
40
0
shLuc
shTac2
B
Anogenital Investigation
ns
50
40
30
20
10
0
C
Body Investigation
*
80
60
40
20
0
D
Attack
60
40
20
0
Females
E
Face Interaction
100
60
F
Anogenital Investigation
125
100
75
50
25
G
Body Investigation
160
120
H
Attack
Verification of Tac2 Knockdown
I
AAV2-CMV-shTac2-zsGreen
OR
AAV2-CMV-shLuciferase-zsGreen
3 weeks
isolation
Resident Intruder
FISH for Tac2
J
Tac2
DAPI
K
% total cells expressing Tac2
5
4
3
2
1

Supplemental Figure 5. Sex-dependent effects of knockdown of Tac2 in mPFC of isolated mice; corresponds to Fig 5

A-D. Males.
A. Total duration of aggression in isolated males with the shRNA shLuciferase (shLuc) or the shTac2 in the mPFC, Mann-Whitney test, $p > 0.05$.
B. Same as A, but total duration of face investigation, Mann-Whitney test, $p > 0.05$.
C. Same as A, but total duration of anogenital investigation, unpaired t-test, $p > 0.05$.
D. Same as A, but total duration of body investigation, unpaired t-test, $p < 0.1$.
E-H. Females.
E. Graph showing the total duration of body investigation in shLuc and shTac2 isolated females, Mann-Whitney test, $p > 0.05$.
F. Same as E, but showing total duration of face threat, Mann-Whitney test, $p > 0.05$.
G. Same as E, but showing total duration of anogenital investigation, Mann-Whitney test, $p < 0.1$.
H. Same as E, but showing the total duration of body investigation, Mann-Whitney test, $p > 0.05$.
I. Workflow of experiment with FISH verification of Tac2 knockdown.
J. Representative images of FISH from animals infused with shLuciferase (shLuc) and shTac2 viruses. Scale represents 50µm.
K. Verification of Tac2 knockdown via RNAscope for *Tac2* in mPFC. Nested t test, $p < 0.05$.

*Bars represent mean ± SEM, individual dots represent mean of individual animals for behavioral data and individual sections for FISH data; ns $p > 0.05$, * $p < 0.05$*

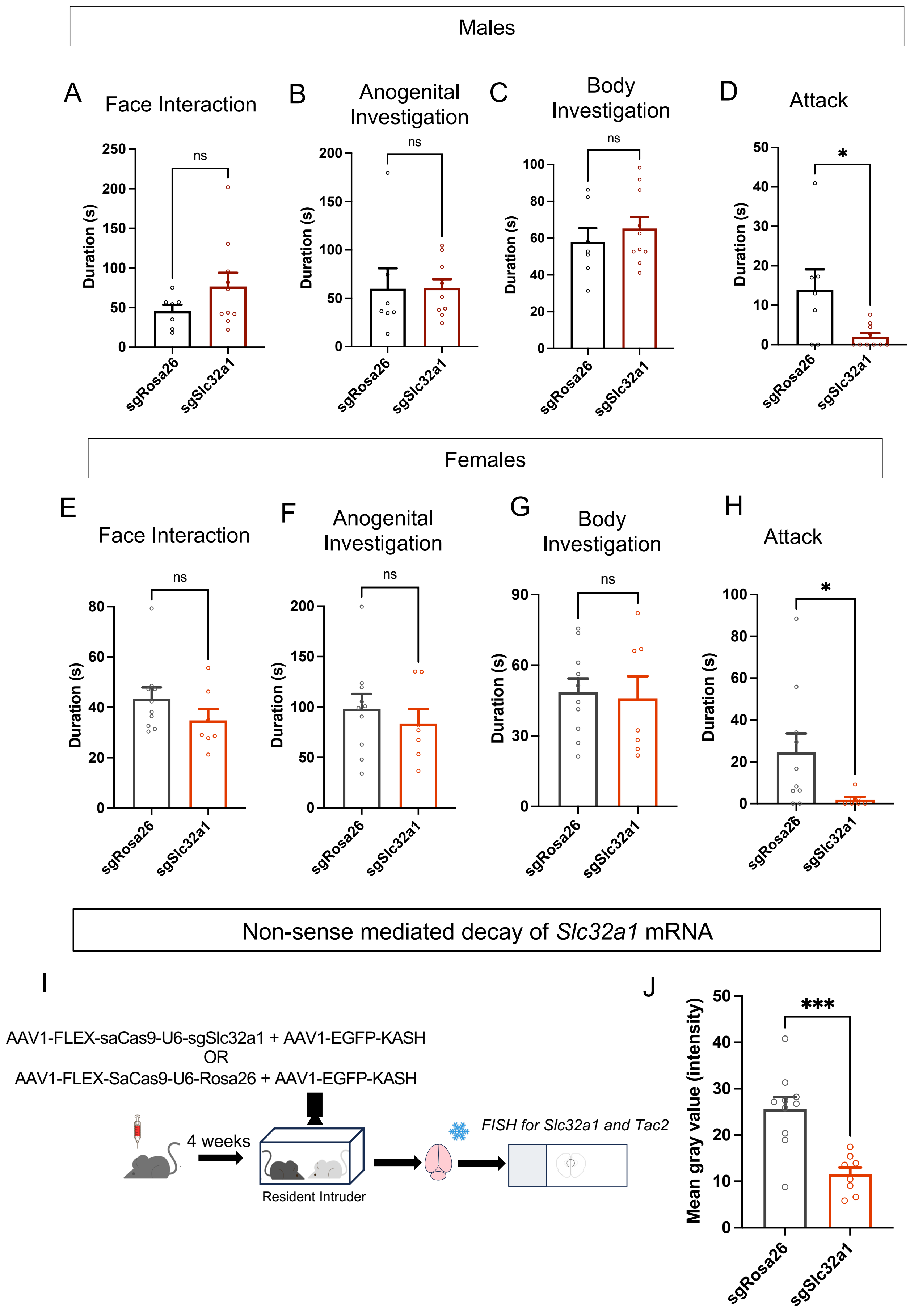

Males
A
Face Interaction
Duration (s)
250
200
150
100
50
0
ns
sgRosa26
sgSlc32a1
B
Anogenital Investigation
Duration (s)
200
150
100
50
0
ns
sgRosa26
sgSlc32a1
C
Body Investigation
Duration (s)
100
80
60
40
20
0
ns
sgRosa26
sgSlc32a1
D
Attack
Duration (s)
50
40
30
20
10
0
*
sgRosa26
sgSlc32a1
Females
E
Face Interaction
Duration (s)
80
60
40
20
0
ns
sgRosa26
sgSlc32a1
F
Anogenital Investigation
Duration (s)
200
150
100
50
0
ns
sgRosa26
sgSlc32a1
G
Body Investigation
Duration (s)
90
60
30
0
ns
sgRosa26
sgSlc32a1
H
Attack
Duration (s)
100
80
60
40
20
0
*
sgRosa26
sgSlc32a1
Non-sense mediated decay of *Slc32a1* mRNA
I
AAV1-FLEX-saCas9-U6-sgSlc32a1 + AAV1-EGFP-KASH
OR
AAV1-FLEX-SaCas9-U6-Rosa26 + AAV1-EGFP-KASH
4 weeks
Resident Intruder
FISH for Slc32a1 and Tac2
J
Mean gray value (intensity)
50
40
30
20
10
0
***
sgRosa26
sgSlc32a1

Supplemental Figure 6. Sex-independent effects of *Slc32a1* missense mutations in mPFC Tac2 neurons of isolated mice; corresponds to Fig 6

A-B. Males.
A. Total duration of aggression in isolated males with CRISPR/SaCas9 virus directed to the Rosa26 locus (sgRosa26) or to *Slc32a1* (sgSlc32a1); Mann-Whitney test, $p < 0.05$.
B. Same as A, but showing the total duration of face investigation, unpaired t-test, $p > 0.05$. **C**. Same as A, but showing the total duration of anogenital investigation, Mann-Whitney test, $p > 0.05$.
D. Same as A, but showing the total duration of body investigation, unpaired t-test, $p > 0.05$.
E-F. Females.
E. Graph showing the total duration of aggression in isolated females with CRISPR/SaCas9 virus directed to the Rosa26 locus (sgRosa26) or to Slc32a1 (sgSlc32a1), Mann-Whitney test, $p < 0.05$.
F. Same as E, but showing the total duration of face threat, Mann-Whitney test $p > 0.05$. **G**. Same as E, but showing the total duration of anogenital investigation, unpaired t- test $p > 0.05$.
H. Same as E, but showing the total duration of body investigation, unpaired t-test, $p > 0.05$.
I. Fluorescence intensity (mean grey value) of Slc32a1 fluorescently labeled mRNA in a mask of $Tac2^{+}$ cells from in situ hybridization, N = 1 F per viral condition, ~ 4 sections per animal, unpaired t-test $p < 0.001$.

*Bars are mean ± SEM, individual dots in A-H represent mean duration for an individual animal. Dots in I represent mean grey value in the one hemisphere of infralimbic cortex of each section. ns p > 0.05, * p < 0.05, ** p < 0.01, *** p < 0.001*
